# Osteogenic differentiation and proliferation potentials of human bone marrow and umbilical cord-derived mesenchymal stem cells on the 3D-printed hydroxyapatite scaffolds

**DOI:** 10.1038/s41598-022-24160-2

**Published:** 2022-11-14

**Authors:** Ladda Meesuk, Jintamai Suwanprateeb, Faungchat Thammarakcharoen, Chairat Tantrawatpan, Pakpoom Kheolamai, Iyapa Palang, Duangrat Tantikanlayaporn, Sirikul Manochantr

**Affiliations:** 1 Division of Cell Biology, Department of Preclinical Sciences, Faculty of Medicine, Thammasat University, Pathumthani, 12120 Thailand; 2 Biofunctional Materials and Devices Research Group, National Metal and Materials Technology Center (MTEC), National Science and Technology Development Agency (NSTDA), Pathumthani, 12120 Thailand; 3 Center of Excellence in Stem Cell Research, Thammasat University, Pathumthani, 12120 Thailand

**Keywords:** Cell biology, Molecular biology, Stem cells

## Abstract

Mesenchymal stem cells (MSCs) are a promising candidate for bone repair. However, the maintenance of MSCs injected into the bone injury site remains inefficient. A potential approach is to develop a bone-liked platform that incorporates MSCs into a biocompatible 3D scaffold to facilitate bone grafting into the desired location. Bone tissue engineering is a multistep process that requires optimizing several variables, including the source of cells, osteogenic stimulation factors, and scaffold properties. This study aims to evaluate the proliferation and osteogenic differentiation potentials of MSCs cultured on 2 types of 3D-printed hydroxyapatite, including a 3D-printed HA and biomimetic calcium phosphate-coated 3D-printed HA. MSCs from bone marrow (BM-MSCs) and umbilical cord (UC-MSCs) were cultured on the 3D-printed HA and coated 3D-printed HA. Scanning electron microscopy and immunofluorescence staining were used to examine the characteristics and the attachment of MSCs to the scaffolds. Additionally, the cell proliferation was monitored, and the ability of cells to differentiate into osteoblast was assessed using alkaline phosphatase (ALP) activity and osteogenic gene expression. The BM-MSCs and UC-MSCs attached to a plastic culture plate with a spindle-shaped morphology exhibited an immunophenotype consistent with the characteristics of MSCs. Both MSC types could attach and survive on the 3D-printed HA and coated 3D-printed HA scaffolds. The MSCs cultured on these scaffolds displayed sufficient osteoblastic differentiation capacity, as evidenced by increased ALP activity and the expression of osteogenic genes and proteins compared to the control. Interestingly, MSCs grown on coated 3D-printed HA exhibited a higher ALP activity and osteogenic gene expression than those cultured on the 3D-printed HA. The finding indicated that BM-MSCs and UC-MSCs cultured on the 3D-printed HA and coated 3D-printed HA scaffolds could proliferate and differentiate into osteoblasts. Thus, the HA scaffolds could provide a suitable and favorable environment for the 3D culture of MSCs in bone tissue engineering. Additionally, biomimetic coating with octacalcium phosphate may improve the biocompatibility of the bone regeneration scaffold.

## Introduction

The prevalence of bone disorders characterized by impaired bone function, such as osteoarthritis, osteomyelitis, and osteoporosis, has grown dramatically globally, particularly among the elderly. Bone tissue disorders are among the most common problems in orthopedics^1^, with substantial clinical and economic impacts^2^. Despite advanced treatment options, the number of patients suffering from failed treatments of the fractured bone, resulting in delayed-union or even nonunion bone, continues to rise. Furthermore, interfering with fracture healing might result in a critical-bone deficiency that requires bone replacement^3^. Autologous bone grafts are often used to treat significant bone defects^4^. While autologous bone grafting is the preferred treatment option for a severe bone defect, it does have some drawbacks, including limited supply, donor-site morbidity, and additional damage to the patient^5^.

On the other hand, although allogeneic bone grafts are widely accessible, they have a substantial risk of infectious disease transmission and rejection by the host immune system responses^6^. To overcome these constraints, alternative approaches using synthetic bone grafts are critical for lowering the hazards and costs associated with autografts and allografts^7^. Thus, bone tissue engineering is promising for developing synthetic bone grafts^6^. It is, nevertheless, a complicated and dynamic process that requires the synergistic actions of artificially constructed extracellular matrix scaffolds, the selection of appropriate stem cells, and a combination of osteogenic stimulating factors^8^. Biomaterials for the artificial bone matrix are usually porous, allowing for cell adhesion and proliferation in three dimensions and the capacity to fill bone defects while providing mechanical support during bone repair and regeneration^9^. Biomaterials for bone tissue engineering must display certain features, including biocompatibility, osteoinduction, and osteoconduction, to be safe and effective in a clinical context^10^.

Numerous synthetic and natural polymers, including hydroxyapatite, collagen, chitosan, and peptide hydrogels, are employed to construct the scaffolds^1^. Hydroxyapatite (HA) is an excellent scaffold material for bone regeneration because it is the main mineral component found in natural bone, and, more importantly, it is biocompatible^11^. Furthermore, it has osteoconductive and osteoinductive properties, as shown by the ability of bone-forming cells in the grafting area to migrate across a scaffold and gradually replace it with new bone over time, as well as its capacity to promote new bone formation in vivo^12^. HA has been used to construct bone grafts in a clinical setting^13^. However, the conventional HA scaffolds are made into the desired shape using ceramic techniques that require high-temperature sintering to burn out the binders and densify the structure. As a result, the high crystallinity and large crystal size restricted the resorption of the scaffold material in vivo, which hindered its integration with a natural bone of the host^14,15^. To circumvent this limitation, three-dimensional printed hydroxyapatite (3D-printed HA) was developed by our team for use as a novel bone graft material^16^. In our study, the novel binder-jetting technique was combined with the low-temperature phosphorization to produce hydroxyapatite with low crystallinity and nano-sized crystals closely resembling the natural hydroxyapatite crystal structure in natural bone. Moreover, our 3D-printed HA also has high porosity and liquid-wicking ability, essential for promoting cell adhesion and other biological activities. 3D printing would also enable a new means to tailor a three-dimensional calcium phosphate scaffold or bone graft with customized shape and structure.

Furthermore, it was considered that the bioactivity of this 3D-printed HA could be further improved. Biomimetic coating with octacalcium phosphate (OCP, Ca_8_H_2_(PO_4_)_6_·5H_2_O) crystals, which mimicked the deposition and mineralization processes that occur in natural bone by using an accelerated calcium phosphate solution (ACS) was also recently developed by our group^17–20^. Thus, OCP crystals were chosen to coat the 3D-printed HA scaffold (coated 3D-printed HA)^20^ to enhance bioactivities due to its superior resorbability, osteoconductivity, osteoinductivity, and binding capacity to biologically active osteogenic stimulating factors compared to hydroxyapatite alone^21–24^. Both 3D-printed HA and coated 3D-printed HA have already been characterized for osteoblastic response and showed promising results. However, the interaction with mesenchymal stem cells has not been carried out yet.

Mesenchymal stem cells (MSCs) are multipotent cells that can differentiate into various mesodermal cell types. They play a critical role in bone modeling, remodeling, and repair^25^. MSCs have shown therapeutic use in orthopedics and regenerative medicine^26,27^. However, transplantation of MSCs to repair bone injury is ineffective owing to the inefficient localization of the cells to the injury site; therefore, maintaining the transplanting MSCs in the targeted site using a scaffold is essential. Recently, MSCs derived from bone marrow (BM-MSCs) cultivated on a three-dimensional scaffold have been used to repair bone tissue. However, the use of BM-MSCs is restricted by various factors, including the invasive procedure for harvesting bone marrow and the limited amount of MSCs in bone marrow, particularly in the elderly^28^. Invariably, the amount of MSCs obtained from bone marrow is less than the critical quantity of cells needed for bone repair^26^.

In addition, both the availability of the cells and the potential for osteogenic differentiation diminish with age, resulting in insufficient bone tissue restoration in the elderly^29^. Besides, there is an accumulation of senescent MSCs and their progeny in the old bone marrow^30–32^. Alternatively, MSCs can be obtained from other tissues, including adipose tissue, placenta, and umbilical cord^33–37^.

To construct a better-quality bone graft, we combined our 3D-printed HA scaffolds with the umbilical cord MSCs (UC-MSCs), which can be harvested in large quantities using a non-invasive procedure and have been considered a more suitable source of MSCs than BM-MSCs^35,38,39^. This study aims to evaluate the effect of coating with biomimetic calcium phosphate on the attachment, proliferation, and osteogenic differentiation of MSC on 3D-printed hydroxyapatite scaffolds. This was tested using two types of MSCs, BM-MSCs and UC-MSCs. Therefore, this study provided novel and valuable information regarding the growth and osteogenic differentiation of human MSCs on 3D-printed HA and coated 3D-printed HA that can be used to develop better scaffolds for bone tissue engineering.

## Materials and methods

### Fabrication of 3D-printed hydroxyapatite scaffold

The 3D-printed hydroxyapatite scaffolds (3D-printed HA) were fabricated as described previously^16^. First, calcium sulfate-based powder (Visijet PXL, 3D Systems, USA) was loaded into the powder-based three-dimensional printing machine (PROJET 160, 3D Systems, USA) to print the closed disc-shaped specimens with 5 mm in diameter and 2 mm in thickness without any designed pore channels in the specimen using a commercial liquid binder (Visijet PXL clear, 3D Systems, USA). Regarding the printing parameters, the layer thickness was set at 0.1 mm, and the disc sample was oriented to build the thickness in the Z direction. Next, the printed samples were phase transformed to hydroxyapatite using the dissolution–precipitation principle by immersing them in a 1 M disodium hydrogen phosphate solution (Sigma Aldrich, USA) at 100 °C for 24 h. Afterward, the scaffolds were cleaned with distilled water, oven-dried, and sterilized using an ethylene oxide sterilizer.

### Biomimetic coated 3D-printed hydroxyapatite scaffold

The biomimetic coated 3D-printed hydroxyapatite scaffolds (coated 3D-printed HA) were done by soaking the prepared 3D-printed HA in an accelerated calcium phosphate solution (ACS) containing 154 mM Na^+^, 201.7 mM Cl^−^, 3.87 mM Ca^2+^, and 2.32 mM HPO_4_^2−^; pH7.3 at 37 °C for 8 h^20^. The coated HA was then gently cleaned, dried overnight at room temperature, and sterilized using an ethylene oxide sterilizer.

### Materials characterizations

The microstructure of the fabricated samples were observed using scanning electron microscopy (JEOL JSM 7800 Prime, Japan). Porosity and pore size were determined by a mercury porosimeter (AutoPore V 9600, Micromeritics Instrument Cooperation, USA) using pressure between 0.10 and 61,000 psia at 20–21 °C. Next, the samples' phase composition was examined using an X-ray diffractometer (XRD, Rigaku TTRAX III, USA) with Cu source Kα line focused radiation (λ = 0.15406 nm) operating at 300 mA and 50 kV. The measurement was conducted at 2–42° 2θ using a scan speed of 3° per minute and a step angle of 0.02°. The XRD pattern was then analyzed for phase composition using the powder diffraction file (PDF), which allowed searching for the ICDD database product. The phase content percentage was then calculated using the Rietveld refinement method (JADE software).

### MSC isolation and culture

This study was approved by the Human Ethics Committee of Thammasat University No. 1 (Faculty of Medicine). Human bone marrows were obtained from 5 healthy donors (27-year-old male, 46-year-old male, 26-year-old female, 42-year-old female, and 53-year-old female). For each representative donor, 10 ml bone marrow was diluted 1:1 with 1X phosphate buffer saline (PBS) and gently placed over IsoPrep (Robbins Scientific Corporation, Norway). Mononuclear cells (MNCs) were recovered from the interphase layer and washed twice with PBS after 30 min of density gradient centrifugation at 100xg (Hettich, Universal 320 K, USA). The cells were then cultured at a density of 1 × 10^5^ cells/cm^2^ in 25-cm^2^ tissue culture flasks (Costa, Corning, USA) in the complete DMEM medium [Dulbecco's Modified Eagle's Medium (DMEM; Gibco/Thermo Fisher Scientific, USA) supplemented with 10% fetal bovine serum (FBS; Hyclone, USA), 2 mM Glutamax™ (Gibco/Thermo Fisher Scientific, USA), 100 U/ml penicillin and 100 µg/ml streptomycin]. The culture was maintained at 37 °C in a humidified tissue culture incubator with 5% carbon dioxide (CO_2_). On day 3, non-adherent MNCs were removed, and the fresh medium was added.

Human umbilical cords were obtained from healthy full-term newborns after receiving written informed consent from their mothers. (The maternal ages are 25-year-old, 29-year-old, 30-year-old, 34-year-old, and 36-year-old, respectively). For each representative donor, umbilical cords (length 2–4 cm) were washed thoroughly with PBS after being acquired from pregnant women after normal delivery. After removing the umbilical cord vessels, the umbilical cord tissue was chopped into small pieces of approximately 1–2 mm^3^. Subsequently, the tissues were washed twice with PBS and incubated with 0.5% trypsin–EDTA (Gibco/Thermo Fisher Scientific, USA) for 3 h at 37 °C with continuous shaking for partial digestion of umbilical cord tissue and release of the cells. To neutralize the trypsin, 10 ml of culture media supplemented with 10% FBS was added. After centrifugation, the supernatant was discarded, and partially digested tissues were transferred to a 25-cm^2^ culture flask containing 5 ml of the complete DMEM medium. The culture was maintained in a 5% CO_2_ incubator, and the culture medium was replaced every 3 days. The flask was carefully examined by an inverted light microscope (Nikon ECLIPSE Ts2R, Japan) to observe the outgrown cells and detect bacterial or fungal contamination.

The attached cells with fibroblastic morphology isolated from each bone marrow and umbilical cord were labeled as passage 0 (P0). For further expansion, P0 cells that had reached a confluence of 80–90% were sub-cultured using 0.25% trypsin-EDTA and re-plating at a density of 1 × 10^4^ cells/cm^2^. In subsequent experiments, the MSCs from each representative donor were examined, and the data were analyzed as a mean of each representative donor.

### Immunophenotype characterization

The expression of cell surface markers in the cells from each donor was assessed. First, the cells in passages 3–5 were removed using 0.25% trypsin–EDTA. Then, 5 × 10^5^ MSCs were resuspended in 50 µl of PBS and treated for 30 min at 4 °C with 5 µl of fluorescein isothiocyanate (FITC) or phycoerythrin (PE)-conjugated antibody against CD34 (Biolegend, USA), CD45 (Biolegend, USA), CD73 (Biolegend, USA), CD90 (Biolegend, USA) and CD105 (Biolegend, USA). After being washed with PBS, the cells were fixed in 1% paraformaldehyde in PBS. Approximately 2 × 10^4^ labeled cells were acquired with flow cytometry (FACScalibur™, Becton Dickinson, USA). The data were analyzed using CellQuest® (Becton Dickinson, USA).

### Adipogenic differentiation assay

MSCs at passages 3–5 from each donor were trypsinized and cultured with a complete DMEM medium in a 35-mm^2^ dish (Costar, Corning, USA) at a density of 5 × 10^3^ cells/cm^2^ overnight to assess their adipogenic differentiation potential. After washing with PBS, adipogenic induction medium [complete DMEM medium supplemented with 0.5 mM isobutylmethylxanthine (Sigma-Aldrich, USA), 1 μM dexamethasone (Sigma-Aldrich, USA), 10 μM insulin (Sigma-Aldrich, USA), 100 μM indomethacin (Sigma-Aldrich, USA)] was added. The MSCs cultured in a complete DMEM medium were used as a control. The cells were maintained in a 5% CO_2_ incubator at 37 °C for 28 days. Then, the cells were fixed with a vapor of 37% formaldehyde at room temperature for 10 min. Finally, the cells were examined under an inverted microscope after staining with 0.3% Oil Red O (Sigma-Aldrich, USA) in 60% isopropanol for 20 min.

### Osteogenic differentiation assay

To investigate the osteogenic differentiation potential of MSCs, the MSCs from each donor at passages 3–5 were trypsinized and cultured with a complete DMEM medium in a 35-mm^2^ dish at a density of 5 × 10^3^ cells/cm^2^ overnight. Subsequently, the cells were washed with PBS, and osteogenic induction medium [complete DMEM medium supplemented with 100 nM dexamethasone, 10 mM β-glycerophosphate (Sigma-Aldrich, USA), and 50 µg/ml ascorbic acid (Sigma-Aldrich, USA)] was added. The cells cultured with a complete DMEM medium were used as a control. After culturing for 28 days, the cells were fixed with 4% paraformaldehyde at 4 °C for 20 min. Next, the fixed cells were stained with 40 mM Alizarin Red S (Sigma-Aldrich, USA) for 30 min at room temperature and observed under an inverted microscope.

### Chondrogenic differentiation assay

To examine the chondrogenic differentiation potential of the isolated MSCs, the MSCs from each donor were trypsinized and plated at a density of 3 × 10^6^ cells/cm^2^ in a 96-well U-bottom plate (Jet Biofil, China) containing a completed DMEM medium. The cells were incubated at 37 °C in a humidified atmosphere with 5% CO_2_ overnight. After removing a medium, the complete MSCgo™ Chondrogenic XF medium (Sartorius, Germany) was added. The medium was replaced every 3 days, and the culture was maintained at 37 °C in a humidified atmosphere with 5% CO_2_ for 14 days. The spheroidal mass was fixed with 10% formalin solution and stained with 1% Alcian Blue in 0.1 N HCl at room temperature overnight. The staining solution was removed, and the spheroidal mass was washed with 0.1 N HCl, 3 times. The stained mass was examined under inverted microscopy. MSCs cultured in a completed DMEM medium served as a control and were treated in the same manner as those cultured in a chondrogenic differentiation medium.

### Assessment of MSC growth

The growth characteristics of BM-MSCs and UC-MSCs were assessed by trypsinizing culture-expanded MSCs (passage 3–5) and re-plating them on a 24-well cell culture plate (Costar, Corning, USA) containing 1 ml of complete DMEM medium at 5 × 10^2^ cells/cm^2^. The cells were maintained at 37 °C in a 5% CO_2_ incubator. Every 2 days, the cells were counted using a hemocytometer. In order to generate a growth curve, the mean of the triplicate cell counts for each day was calculated and plotted against culture time.

To measure the growth kinetics of MSCs, the cells from passages 2–8 were seeded onto a 24-well cell culture plate at a density of 5 × 10^2^ cells/cm^2^. The cells from each passage were counted every 48 h to calculate the doubling time according to the following formula:

$${\text{Population}}\;{\text{doubling}}\;{\text{time = }}\frac{{{\text{Cell}}\;{\text{culture}}\;{\text{time}}\left( {\text{h}} \right)}}{{{\text{Log}}\left( {\frac{{{\text{Number}}\;{\text{of}}\;{\text{cells}}\;{\text{at}}\;{\text{the}}\;{\text{end}}}}{{{\text{Number}}\;{\text{of}}\;{\text{cells}}\;{\text{at}}\;{\text{day}}\;0}}} \right){ \times }3.31}}$$

### Actin filament staining

To seed cells, the sterile 3D-printed HA/ coated 3D-printed HA were washed twice with PBS, incubated with a complete DMEM medium, and finally put into a 96-well plate (Corning costa, USA). The MSCs (2 × 10^4^ cells) were seeded on the surface of 5-mm scaffold. The cultures were maintained in a complete DMEM medium at 37 °C in a 5% CO_2_ incubator. For assessment of cell attachment on the scaffolds, the actin filament was visualized using Phalloidin-iFluor 488 staining (Abcam, UK). Briefly, the samples were washed with PBS 3 times and fixed with 4% paraformaldehyde at room temperature for 30 min. After careful aspiration of a fixative solution, the samples were washed in triplicate with PBS. Subsequently, the cells were permeabilized with 0.1% Triton X-100 (USB Corporation, USA) at room temperature for 5 min and blocked with 1% BSA for 60 min. Then, the cells were incubated with Phalloidin-iFluor 488 reagent diluted with 1% BSA in PBS at a dilution of 1:1000 at room temperature for 60 min. Next, the cells were gently rinsed with PBS 3 times to remove the excess phalloidin. Finally, the nucleus was visualized by staining with 4, 6‐diamidino‐2‐phenylindole (DAPI) (Sigma-Aldrich, USA). The samples were observed under a confocal microscope (Confocal Microscope System C1; Nikon, Japan) at an excitation of 493 nm and an emission of 517 nm.

### Observations of cell morphology

To examine the morphology of seeded cells, MSCs (2 × 10^4^ cells) were seeded on the surfaces of 5-mm scaffold in a 96-well plate containing a complete DMEM medium or osteogenic differentiation medium. The cells were kept in a humidified incubator with 5% CO_2_ at 37 °C. The medium was changed every 3 days. On day 28, the samples were washed twice with PBS and fixed with 4% glutaraldehyde plus 2% paraformaldehyde in 0.1 M Millonig's buffer pH7.2 at 4 °C for 60 min. Then, the samples were washed 3 times with 0.1 M Millonig's buffer, followed by post-fixation in 1% osmium tetroxide in 0.1 M Millonig's buffer for 30 min. Then, the samples were rewashed in 0.1 M Millonig's buffer and dehydrated with serial concentrations of ethanol, 50%, 70%, 90%, and 95%, consecutively for 10 min in each concentration. Finally, the samples were dehydrated with 100% ethanol for 15 min, 3 times, followed by critical point drying using liquid CO_2_. The samples were gold-sputtered before observation by scanning electron microscopy (JEOL JSM 7800 Prime, Japan).

### Cell proliferation assessment

To assess the affinity of BM-MSCs and UC-MSCs to the scaffolds, the proliferation of BM-MSCs/ UC-MSCs cultured on the 3D-printed HA/ coated 3D-printed HA was investigated using PrestoBlue™ assay (Thermo Fisher Scientific, USA) according to the manufacturer's protocol. Briefly, the cells in passages 3–5 were removed using 0.25% trypsin-EDTA and trypan blue staining was performed to determine the number of living cells. Viable MSCs (2 × 10^4^ cells) were seeded on the surfaces of the 5-mm scaffold in a 96-well plate containing phenol red-free complete medium [phenol red-free Dulbecco's Modified Eagle's Medium (Gibco/Thermo Fisher Scientific, USA) supplemented with 10% fetal bovine serum (FBS; Hyclone, USA), 2 mM Glutamax™ (Gibco/Thermo Fisher Scientific, USA), 100 U/ml penicillin and 100 µg/ml streptomycin]. The cells were kept at 37 °C in a 5% CO_2_ incubator, and the culture medium was changed every 3 days. To avoid attached cells on the culture plate contributing to the assay and influencing the result, the scaffold was transferred to a new plate after seeding overnight. The cell viability of MSCs on the scaffold was measured on days 3, 7, 14, 21, and 28 using PrestoBlue™ assay in the same well without removing scaffolds from the wells. Briefly, the samples were incubated with a PrestoBlue™ reagent in phenol red-free complete medium at a final dilution of 1:10 ratio for 30 min. Then, 100 µl of the reaction medium was spectrophotometrically measured at 560 nm excitation and 590 nm emission using a microplate reader (BioTex, USA). The results were expressed as a percentage of control according to the following formula:

$${\text{Cell}}\;{\text{proliferation}} = \left( {\frac{{{\text{O}}.{\text{D}}.\;{\text{of}}\;{\text{cells}}\;{\text{in}}\;{\text{each}}\;{\text{day}}{-}{\text{O}}.{\text{D}}.\;{\text{of}}\;{\text{blank}}}}{{{\text{O}}.{\text{D}}.\;{\text{of}}\;{\text{cells}}\;{\text{on}}\;{\text{day}}\;{1}{-}{\text{O}}.{\text{D}}.\;{\text{of}}\;{\text{blank}}}}} \right){ \times }100$$

### Alkaline phosphatase activity assay

To examine the osteogenic differentiation potential of BM-MSCs and UC-MSCs cultured on 3D-printed HA/ coated 3D-printed HA, alkaline phosphatase (ALP) activity was measured using SensoLyte® pNPP ALP assay kit (AnaSpec, USA). Briefly, 2 × 10^4^ MSCs were seeded on 3D-printed HA/ coated 3D-printed HA in a 96-well plate containing phenol red-free complete medium. The cells were allowed to adhere to the surface overnight, and the phenol red-free complete medium was substituted with the osteogenic differentiation medium. The cells were maintained in a humidified incubator at 37 °C with 5% CO_2,_ and the fresh medium was changed every 3 days. ALP activity was determined on days 3, 7, 14, 21, and 28. Briefly, the scaffolds were removed from 96-well plate and placed into a new well. The MSCs on the scaffold were incubated with lysis buffer [0.1 M glycine (VWR Chemicals BDH®, USA), 1% Nonidet P-40 (USB Chemical, USA), 1 mM MgCl_2_ (Sigma-Aldrich, USA), and 1 mM ZnCl_2_ (EMSURE®, Germany), pH 9.6] at room temperature for 20 min. After that, the samples were frozen at − 80 °C for 30 min and thawed at room temperature for 20 min. After a freeze–thaw cycle was repeated 3 times, the samples were centrifuged at 12,000xg, 4 °C for 10 min to sediment cell debris. The supernatant was collected for ALP activity assay using a microplate reader at an absorbance of 405 nm. Total cellular protein was measured using a Bradford assay (Bio-Rad, USA). The ALP activity was calculated by comparing the OD value of the sample (O.D. sample – O.D. blank) with a standard curve generated from 0–10 ng/ml of ALP standard solution and normalized with the total cellular protein concentration.

### Quantitation of osteogenic gene expression

BM-MSCs and UC-MSCs (1 × 10^5^ cells) were cultured on 15-mm 3D-printed HA/ coated 3D-printed HA in a 24-well plate containing the complete DMEM medium overnight. Then, the medium was replaced with an osteogenic differentiation medium. The cells were maintained at 37 °C in a 5% CO_2_ incubator, and the fresh medium was changed every 3 days. Total RNA was extracted using Trizol™ Reagent (Thermo Fisher Scientific Invitrogen, USA) on days 7, 14, 21, and 28. Briefly, MSCs on a scaffold were lysed with 500 μl of Trizol™ reagent. An initial − 80 °C freeze for 30 min followed by a 25 °C thaw for 20 min. The cycle was repeated 3 times. The lysed cells were transferred to a 1.5 ml tube. Subsequently, 100 µl of chloroform was added, and the mixture was vigorously shaken for 15 s before being incubated for 2 min at room temperature. After centrifugation at 12000xg for 15 min at 4 °C, the aqueous phase was transferred to a new tube, and isopropanol was added. The sample was incubated at room temperature for 10 min before centrifugation at 12000xg, 4 °C for 10 min. The supernatant was discarded, and the RNA pellet was washed with 75% ethanol in nuclease-free water and dried at room temperature for 10 min. The RNA was then resuspended in 50 µl of RNAse-free water. The RNA concentration was measured using a nanodrop machine (Thermo Fisher Scientific, USA) and reverse transcribed into cDNA using iScript™ Reverse Transcription Supermix for RT-qPCR (Bio-Rad, USA). The qRT-PCR reactions were prepared using the iTaq™ Universal SYBR® Green Supermix (Bio-rad, USA). The reactions were amplified using StepOne plus™ Real-Time PCR system (Applied Biosystems; ABI, USA.) with 40 cycles of amplification (denaturation at 95 °C for 15 s, annealing at 60 °C for 60 s). The primer sequences, including runt-related transcription factor-2 (*RUNX-2*), osterix (*OSX*), osteocalcin (*OCN*), and glyceraldehyde-3-phosphate dehydrogenase (*GAPDH*), were summarized in Table 1 (Table 1). The differential expressions of the osteogenic genes were normalized by the internal control gene. The data were analyzed by the comparative threshold cycle value (ΔΔCT) method using StepOne™ Software version 2.3 (Applied Biosystems; ABI, USA.) and presented as the relative mRNA expression level.

**Table 1:** The primers and the product size.

Gene	Forward primer	Reverse primer	Product size (bp)
RUNX-2	5′-GACAGCCCCAACTTCCTGTG-3′	5′-CCGGAGCTCAGCAGAATAAT-3′	159
Osterix	5′-TGCTTGAGGAGGAAGTTCAC-3′	5′-CTGCTTTGCCCAGAGTTGTT-3′	114
Osteocalcin	5′-CTCACACTCCTCGCCCTATT-3′	5′-TCAGCCAACTCGTCACAGTC-3′	245
GAPDH	5′-CAATGACCCCTTCATTGACC-3′	5′-TTGATTTTGGAGGGATCTCG-3′	159

### Immunofluorescence staining

The expression of bone matrix collagenous and non-collagenous proteins in BM-MSCs and UC-MSCs cultured on 3D-printed HA/ coated 3D-printed HA were examined using immunofluorescence staining. Briefly, 2 × 10^4^ MSCs were seeded on 3D-printed HA/ coated 3D-printed HA in a 96-well plate containing phenol red-free complete medium. The cells were allowed to adhere to the surface overnight. Then, the complete medium was removed and the osteogenic differentiation medium was added. The cells were maintained in a humidified incubator at 37 °C with 5% CO_2_. The fresh medium was changed every 3 days. Immunofluorescence staining was performed on day 21. Briefly, the MSCs on the scaffold were fixed with 4% paraformaldehyde for 15 min and permeabilized with 0.1% Triton X-100 (USB Corporation, USA) for 10 min. The cells were then washed in PBS and incubated with 4% bovine serum albumin (BSA; Sigma-Aldrich, USA) in PBS at room temperature for 30 min. Subsequently, the cells were incubated with 4% BSA-PBS at room temperature for 30 min. Then the cells were incubated with an anti-osteocalcin antibody (Abcam, UK: ab93876, 1:100) and an anti-collagen I antibody (Abcam, UK: ab34710, 1: 500) at 4 °C overnight. The cells were washed with 0.1% tween 20 in PBS for 5 min 3 times and incubated with Alexa Fluor®488 goat anti-rabbit IgG (H + L) (Thermo Fisher Scientific Inc., USA: A11034, 1:500) for 30 min at room temperature. Nuclei were counterstained with 1 µg/ml of Hoechst (Sigma-Aldrich, USA). The cells were examined using confocal microscopy (Confocal Microscope System C2 + ; Nikon, Japan). The cells cultured in the completed medium were used as controls.

### Statistical analysis

All experiments were conducted on at least 3 distinct samples. The data were presented as mean ± standard deviation (SD). Statistical analysis was performed using one-way analysis (ANOVA). A P-value of less than 0.05 was considered to be statistically significant.

### Ethics approval and consent to participate

This study was approved by the Human Research Ethics Committee of Thammasat University No.1 (Faculty of Medicine) and followed tenets of the declaration of Helsinki and Belmont report. All samples were obtained from donors with written informed consent.

## Results

### Characterization of 3D printed scaffold

**Figure 1:**
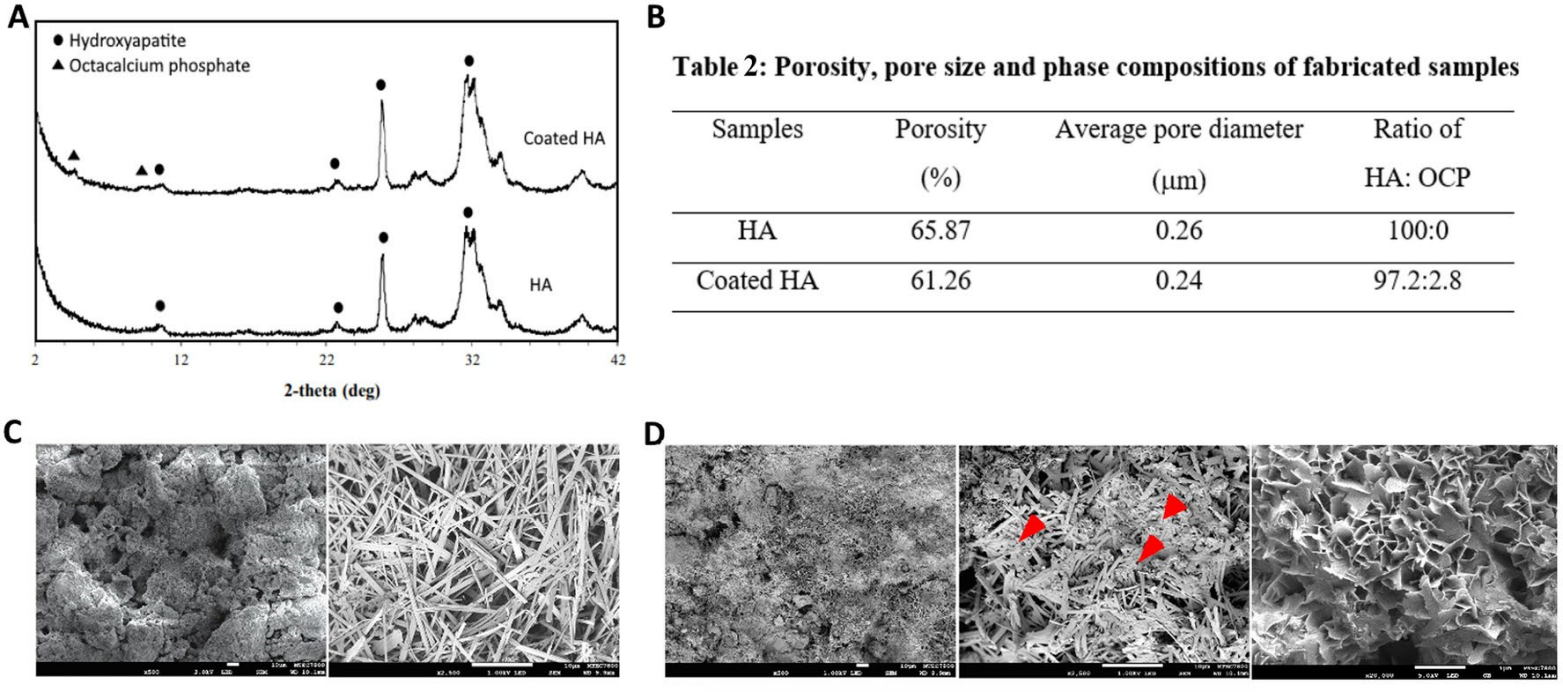
The characteristics of 3D-printed scaffolds. (**A**) The XRD patterns showing the broad peaks of both samples indicate low crystalline nature and a monophasic phase of hydroxyapatite in the HA sample and hydroxyapatite/octacalcium phosphate phases in the coated HA sample. (**B**) Table displaying the porosity, pore size, and phase ratio of HA: OCP of the samples. (**C**) Microstructure of 3D-printed HA (HA) showing a porous nature (left, × 500) which was formed by the entanglement of the needle-like crystals of HA (right, × 2,500). (**D**) Microstructure of coated 3D-printed HA (coated HA) showing a porous structure (left, × 500) comprising a mixture of the needle-like crystals of HA and the OCP crystals as indicated by red arrows (middle, × 2,500), which were leaf-like crystals (right, × 20,000).

The XRD patterns of 3D-printed HA and coated 3D-printed HA have the same prominent peaks at 2θ of 11°, 23°, 26°, and 32°, which were the typical peaks of the HA phase (Fig. 1A). Small peaks at about 5°, and 9°, belonging to octacalcium phosphate (OCP) were also observed in coated 3D-printed HA. This suggested that the biomimetic deposition of OCP onto the HA matrix was successful, with the ratio of HA: OCP in the sample was 97.2:2.8 (Fig. 1B). The porosity of coated 3D-printed HA was slightly lower than that of 3D-printed HA, 61.26% versus 65.87%, whereas the average pore size of both samples was in similar ranges of 0.24 and 0.26 μm (Fig. 1B). By a scanning electron microscopy, the microstructures of 3D-printed HA and coated 3D-printed HA revealed a similar highly porous nature but with distinct crystal morphology. The 3D-printed HA displayed the entanglement of needle-like crystals of hydroxyapatite (Fig. 1C) which were formed during the low-temperature phase transformation process. In contrast, leaf-like crystals of octacalcium phosphate were additionally deposited onto the hydroxyapatite crystals due to the biomimetic coating step in coated 3D-printed HA (Fig. 1D).

### Characterization of mesenchymal stem cells

**Figure 2:**
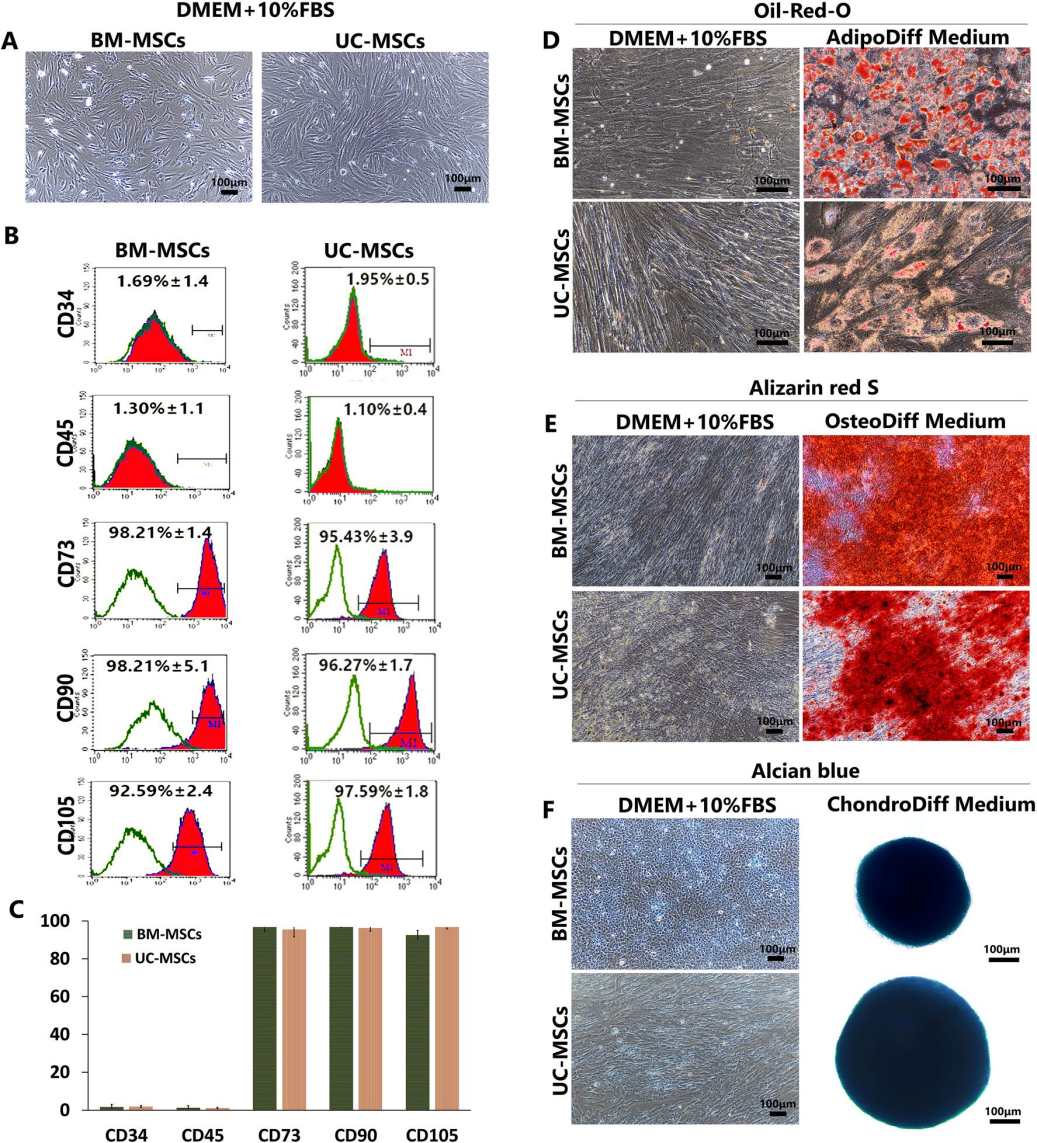
The characteristics of MSCs derived from human bone marrow (BM-MSCs) and umbilical cord (UC-MSCs). (**A**) The spindle morphology of the cells cultured in DMEM + 10%FBS for 10 days. (**B**) Flow cytometry analysis showed the positive expression of MSC markers (CD73, CD90, CD105) and negative expression of hematopoietic markers (CD34, CD45). (**C**) The expression of MSC markers in BM-MSCs and UC-MSCs was not statistically different. Data are presented as mean ± standard deviation (SD). (**D**) The adipogenic differentiation potential of BM-MSCs and UC-MSCs, the differentiated MSCs exhibited orange-red color after staining with Oil Red O. (**E**) The osteogenic differentiation potential of BM-MSCs and UC-MSCs, the differentiated MSCs exhibited orange-red color after staining with Alizarin Red S. (**F**) The chondrogenic differentiation potential of BM-MSCs and UC-MSCs, the differentiated MSCs exhibited blue color after staining with Alcian blue. N = 5, Micron bar = 100 μm.

Mesenchymal stem cells were isolated from human bone marrow (BM-MSCs) using density gradient centrifugation. After seeding, the spherical-shaped cells adhered to the culture flask and exhibited fibroblast-like morphology. On day 3, non-adherent cells were removed, and microscopic examination revealed several colonies of adherent cells. The cells in the colony exhibited a spindle-shaped and fibroblastic appearance (Fig. 2A). The cells had a high proliferation capacity. They were expanded up to 10 passages before reaching a senescence phase.

The cells from the umbilical cord were isolated using enzymatic digestion and cultured in the same condition as those isolated from bone marrow. A day after initial plating, the loosely adherent aggregated cells from the umbilical cord were observed under inverted microscopy. These cells were continually cultured, and 80% confluence of a homogeneous population of fibroblast-like cells was observed within 10 days after initial seeding (Fig. 2A). After passage, UC-MSCs exhibited a rapid proliferative capacity in normal cultured conditions. UC-MSCs could be expanded up to 20 passages before losing their proliferative capacity.

### Immunophenotype characterization

The cultured MSCs at passage 3 to passage 6 were analyzed by flow cytometry to test for the cluster of differentiation (CD) markers, including CD73, CD90, CD105, CD34, and CD45. The results showed that the mean percentage of cells expressing markers characteristic of hematopoietic cells (CD34 and CD45) was overall below 2%. These markers were referred to as exclusion markers. On the contrary, cells isolated from bone marrow and umbilical cord were positive for markers initially referred to as MSC markers (CD73, CD90, and CD105). Notably, more than 90% of BM-MSCs and UC-MSCs were positive for MSC markers (CD73, CD90, and CD105) (Fig. 2B)**.** However, there was no significant difference in MSC surface marker expression levels between BM-MSCs and UC-MSCs (Fig. 2C).

### Trilineage differentiation potential

The trilineage differentiation potentials of BM-MSCs and UC-MSCs were examined by induction in adipogenic, chondrogenic and osteogenic induction media. Under their distinct inductive culture settings, the cultured MSCs readily differentiated into adipogenic, chondrogenic, and osteogenic lineages. In an adipogenic induction medium, the spindle-shaped MSCs developed into giant cells with numerous lipid droplets in their cytoplasm (Fig. 2D). These lipid droplets were positive for Oil Red O staining, which identified the adipogenic differentiation efficiency of the MSCs. Control MSCs cultured in a complete DMEM medium showed no evidence of adipogenic differentiation and were negative for Oil Red O staining (Fig. 2D). To investigate the osteogenic differentiation potential of BM-MSCs and UC-MSCs, the MSCs were cultured in an osteogenic induction medium and stained with Alizarin Red S to detect the secretion of extracellular calcium and phosphate crystals. After exposure to osteogenic induction medium, signs of extracellular matrix mineralization were detected in both BM-MSCs and UC-MSCs, as judged by positive Alizarin Red S staining, while control cells cultured in complete DMEM medium at the same period remained negative for Alizarin Red S staining (Fig. 2E). The chondrogenic differentiation potential of BM-MSCs and UC-MSCs was assessed after induction in a chondrogenic differentiation medium for 14 days. Both BM-MSCs and UC-MSCs formed a pellet at the bottom of the plate during maintenance in the culture, which grew bigger and more spherical with time (Fig. 2F). On day 10, UC-MSCs formed a spherical mass with a diameter of 520.50 ± 14.54 µm, while BM-MSCs had a diameter of 368.57 ± 23.78 µm. On day 14, UC-MSCs formed a spherical mass with a diameter of 561.11 ± 71.31 µm, while BM-MSCs had a diameter of 547.77 ± 91.27 µm. These spherical masses displayed intense Alcian blue staining, consistent with the proteoglycan deposition. The controls, which were grown in a complete DMEM medium, failed to form pellets and did not positive for Alcian blue staining (Fig. 2F).

### Growth characteristics of MSCs

**Figure 3:**
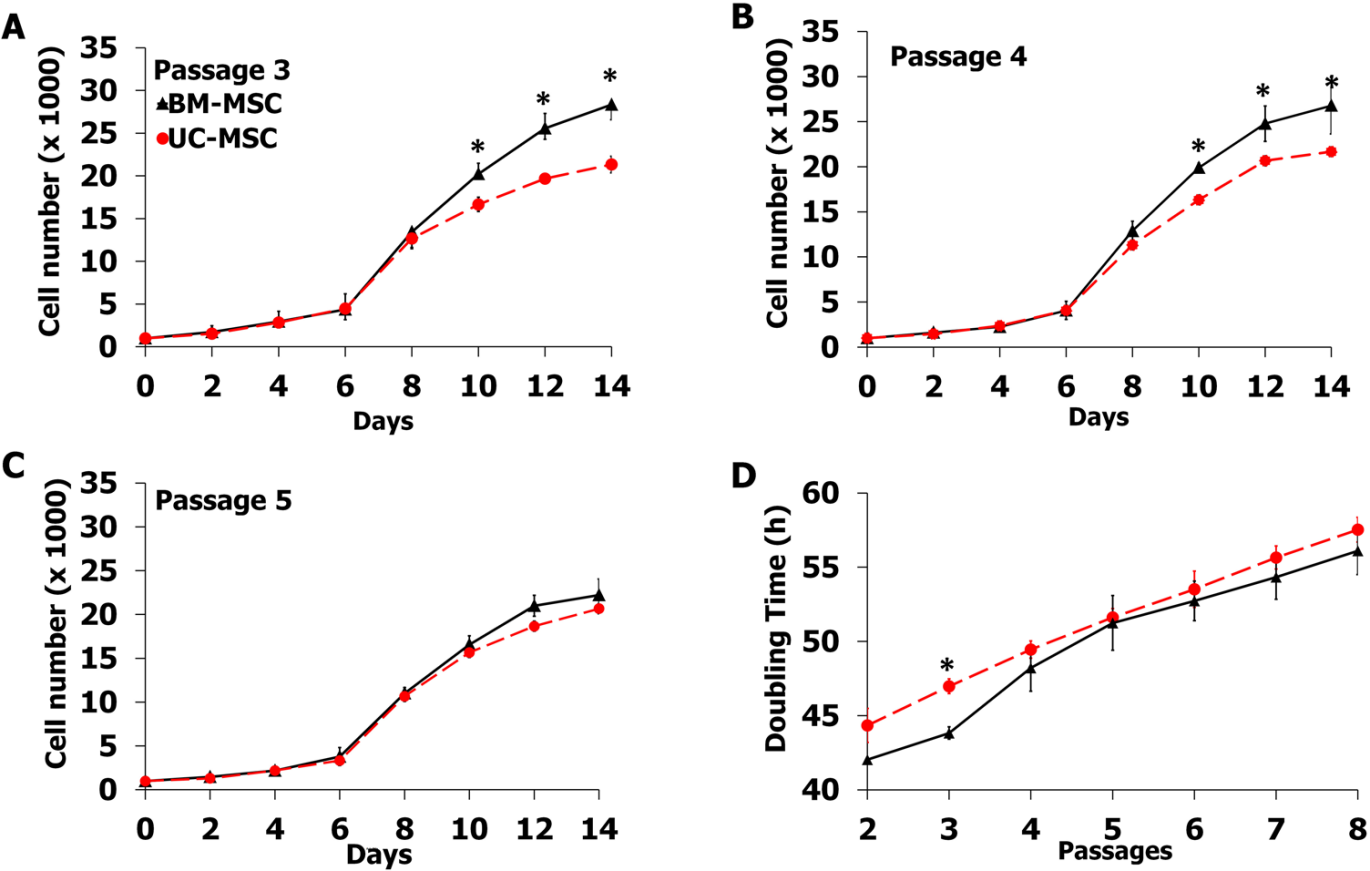
Growth characteristics of UC-MSCs compared to BM-MSCs. Triplicate cultures were harvested every 2 days for 14 days, and adherent cells were counted. (**A**) Growth curve of MSCs at passage 3, (**B**) Growth curve of MSCs at passage 4, (**C**) Growth curve of MSCs at passage 5. (**D**) Population doubling time of UC-MSCs compare to BM-MSCs. Results (N = 3) are expressed as mean ± SD. *P < 0.05: significant difference compared to BM-MSCs.

The growth characteristics of BM-MSCs and UC-MSCs were observed for 14 days in standard culture conditions. The total numbers of the MSCs from each source were evaluated every 2 days using a hemacytometer. During the early period (day 0-day 8), the proliferative capacity of both BM-MSCS and UC-MSCs was similar (Fig. 3A-C). The cell number and growth kinetic during the first 8 days of culture were insignificant. From day 8 onward, the number of UC-MSCs at passages 3-4 was significantly lower than that of BM-MSCs at the same passages (*P* < 0.05). However, it was necessary to note that UC-MSCs were shown to have the same proliferation capacity as BM-MSCs from passage 5 onward. Starting from 1 × 10^3^ cells at day 0, BM-MSCs were expanded for 25 folds within 14 days. On the other hand, UC-MSCs have expanded 20 folds within 14 days (Fig. 3).

The population doubling time of BM-MSCs during passages 2–3 appeared to be significantly lower than those of UC-MSCs (*P* < 0.05), resulting in a more rapid proliferation rate of BM-MSCs than UC-MSCs during the early passage (Fig. 3D). However, the population doubling time of BM-MSCs and UC-MSCs seems to be equal from passage 4 onward. Based on the results, BM-MSCs tended to double their population on an average of 47.79 ± 5.32 h, while UC-MSCs exhibited a doubling time of 51.30 ± 4.71 h.

### Actin filament staining

**Figure 4:**
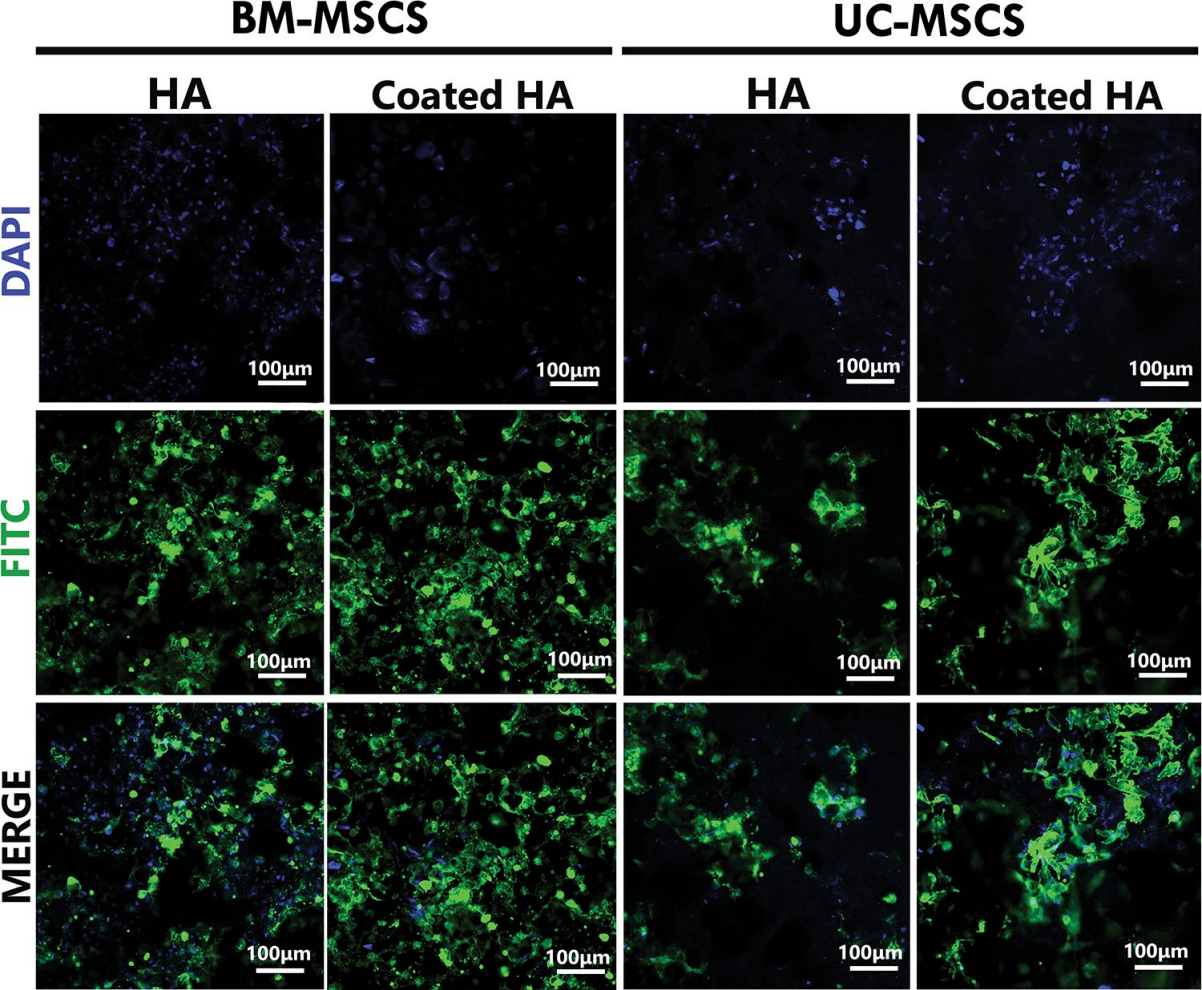
The representative fluorescent micrographs illustrate the actin filament staining (green) in BM-MSCs and UC-MSCs, which attach to the 3D-printed HA (HA) and coated 3D-printed HA (coated HA) on day 28. The nuclei were stained with DAPI (blue).

To evaluate the potential and suitability of 3D-printed HA and coated 3D-printed HA for bone tissue engineering, the adhesion of BM-MSCs and UC-MSCs on the scaffold was observed using actin filament staining. Both 3D-printed HA and coated 3D-printed HA acted as excellent supporting materials for MSCs, as shown in the fluorescent micrographs depicting actin stain (Fig. 4). After initial seeding, both BM-MSCs and UC-MSCs adhered well to the surface of the scaffolds. They formed colonies that covered almost the entire surface of the scaffolds, as evidenced by positive staining of actin filaments within the cells (Fig. 4). Remarkably, both BM-MSCs and UC-MSCs showed more intense staining signals on coated 3D-printed HA than 3D-printed HA, but with no differences in cell shape. Additionally, UC-MSCs cultured on the 3D-printed HA and coated 3D-printed HA appeared as bulky masses of cells on the scaffolds, whereas BM-MSCs appeared as an evenly distributed pattern.

### Characteristic of the cells culture on the 3D scaffolds

**Figure 5:**
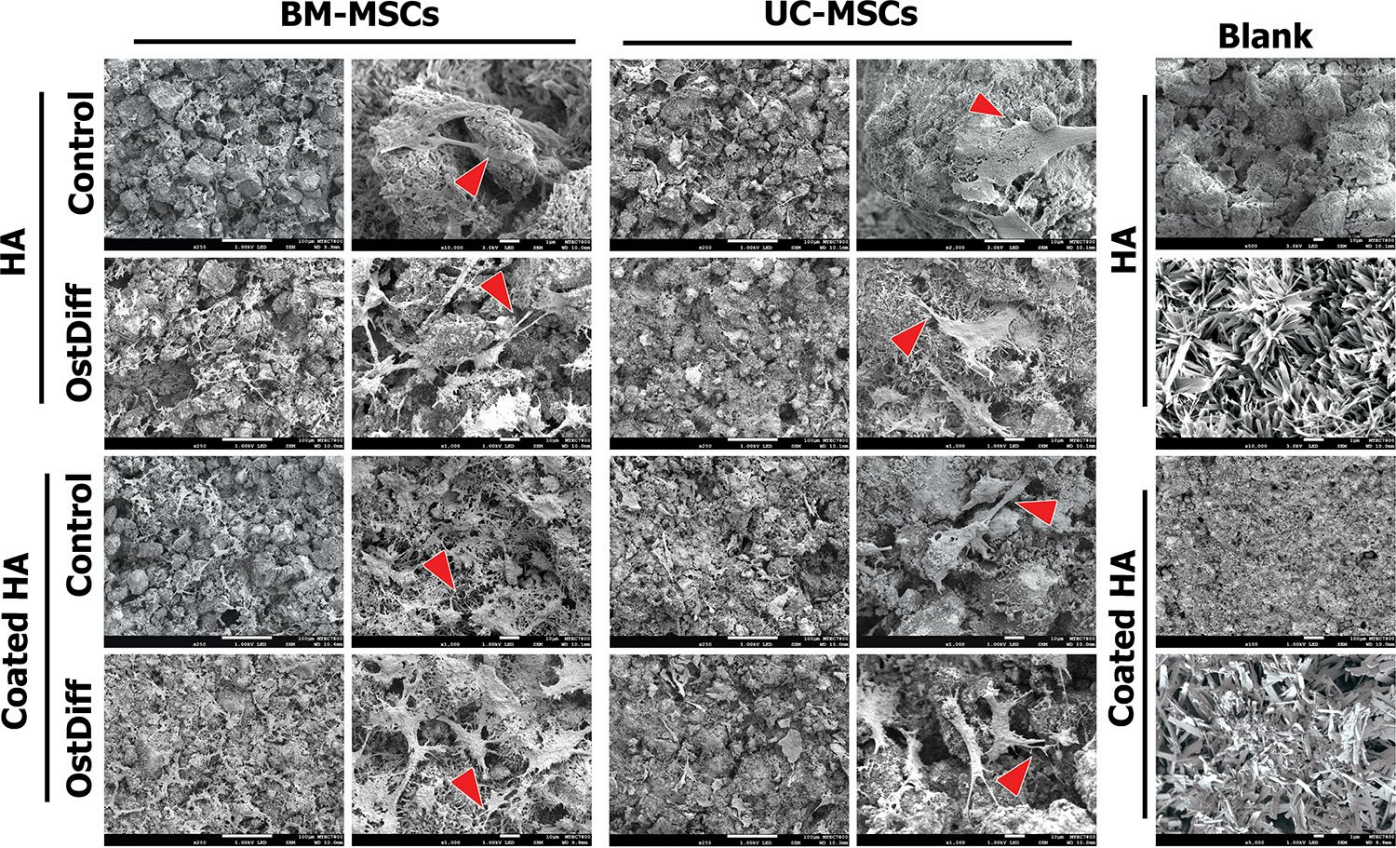
Scanning electron microscopic images demonstrate the characteristics of BM-MSCs and UC-MSCs cultured with osteogenic differentiation medium on 3D-printing HA (HA) and coated 3D-printing HA (coated HA) compared to the cells cultured with complete DMEM medium for 28 days. The cells attach to the scaffolds and become large-flatten cells with extended and interconnected cytoplasmic processes (red arrow).

The morphological characteristics of BM-MSCs and UC-MSCs on the scaffolds were observed on day 28. The BM-MSCs spread well on the surface of 3D-printed HA and coated 3D-printed HA and exhibited a flattened long slender shape with several extending pseudopodia (Fig. 5-red arrow). These pseudopodia appeared connected, and the focal adhesions were formed at the points of contact with the surface of the scaffolds (Fig. 5).

Interestingly, there was a higher density of BM-MSCs cultured in the osteogenic differentiation medium than the BM-MSCs cultured in the completed DMEM medium on both 3D-printed HA and coated 3D-printed HA. The morphological characteristics of UC-MSCs cultured on 3D-printed HA and coated 3D-printed HA were similar to those of BM-MSCs. Both MSCs could remain on the surface of 3D-printed HA and coated 3D-printed HA for at least 28 days while maintaining the flattened cell bodies with long slender shape pseudopodia attached to the surface of the scaffolds (Fig. 5). In addition, the connections between pseudopodia, focal adhesions, and cell bridging leading to cell sheet formation were observed (Fig. 5).

With a prolonged culture period, both BM-MSCs and UC-MSCs exhibited the formation of dense and continuous cell sheets. It was also observed that many MSC colonies were attached to the scaffolds on day 28, with a slightly better outcome on the coated 3D-printed HA. Furthermore, higher densities of both types of MSCs were observed when the samples were cultured in an osteogenic differentiation medium than in the completed DMEM medium, with better results expressed by UC-MSCs. This data might reflect the difference in osteoconductive properties of the scaffolds.

### Cell proliferation assessment

**Figure 6:**
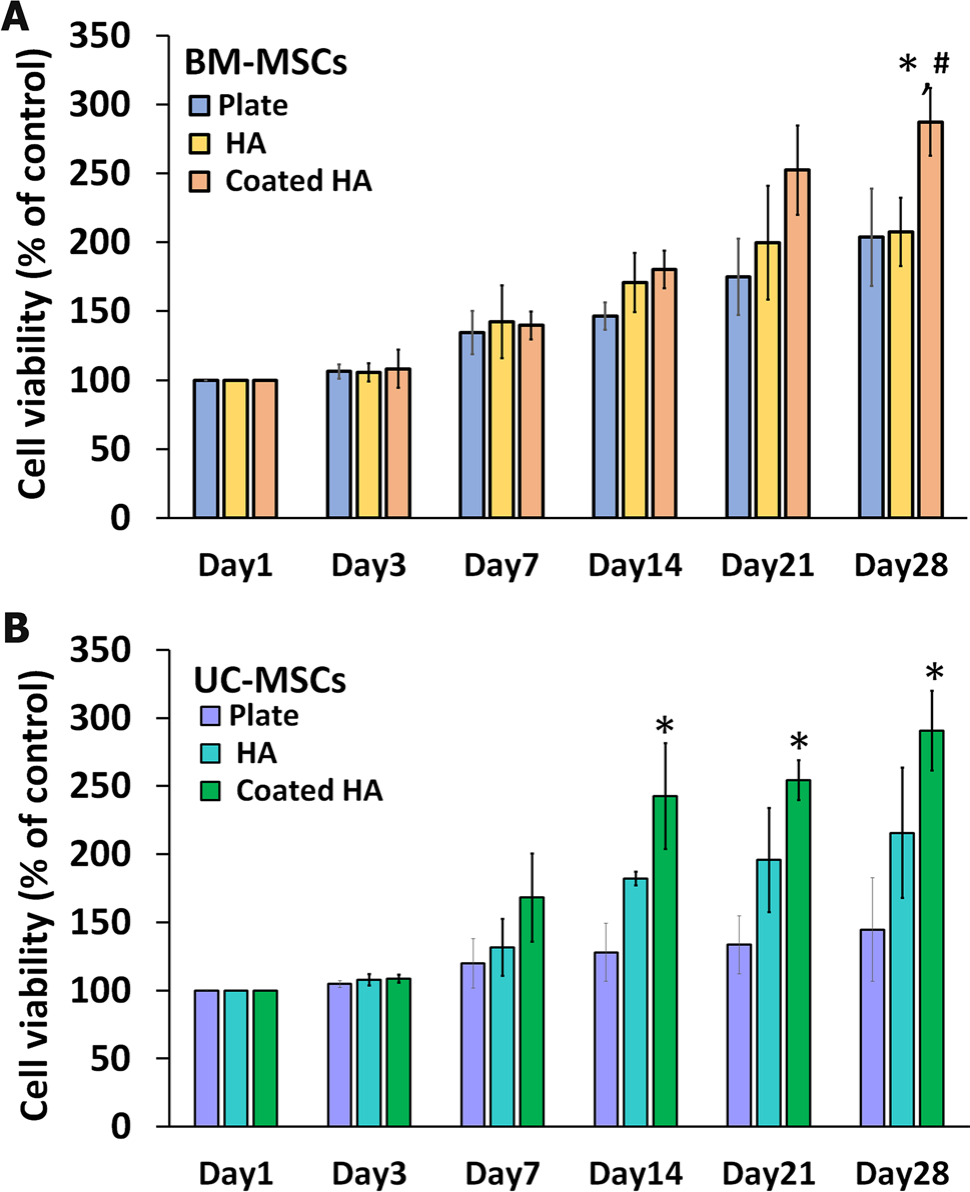
The viability of BM-MSCs (**A**) and UC MSCs (**B**) cultured with complete DMEM medium on the 3D-printed HA (HA), coated 3D-printed HA (coated HA), and plastic culture plate. The viabilities of MSCs in each type of scaffold are presented as % of control which is MSCs cultured on the same type of scaffold on culture day 1. N = 3, *P < 0.05 compared to MSCs cultured on a plastic culture plate. ^#^*P* < 0.05 compared to MSCs cultured on 3D-printed HA.

The biocompatibility of the 3D-printed HA and coated 3D-printed HA was demonstrated using a cell proliferation assay. BM-MSCs and UC-MSCs were cultured on the scaffolds, and the cell proliferation was estimated using the PrestoBlue™ assay. On day 1 after seeding, BM-MSCs on the coated 3D-printed HA had a similar attachment rate to those on the 3D-printed HA (94.2 ± 1.10 vs. 93.9 ± 0.64). BM-MSCs cultured on the plastic culture plate and the scaffolds steadily increased at every time point (Fig. 6A). Interestingly, BM-MSCs cultured on a 3D-printed HA scaffold exhibited a similar degree of cell viability to BM-MSCs cultured on a plastic culture plate. By contrast, BM-MSCs cultured on a coated 3D-printed HA showed significantly higher cell viability than BM-MSCs cultured on a plastic culture plate, especially on day 28 (*P* < 0.05). Significantly, BM-MSCs cultured on a coated 3D-printed HA for day 28 had significantly higher cell viability than those cultured on a 3D-printed HA (*P* < 0.05).

The viability of UC-MSCs cultured on the 3D-printed HA and coated 3D-printed HA compared to those cultured on plastic culture plates showed a similar pattern to BM-MSCs. On day 1 after seeding, UC-MSCs on the coated 3D-printed HA had a similar attachment rate to those on the 3D-printed HA (94.6 ± 1.10 vs. 94.0 ± 1.34). There was a gradual increase in cell viability cultured on the plastic culture plate and the scaffolds throughout the cultured period (Fig. 6B). Nevertheless, UC-MSCs cultured on the plastic culture plate had a lower accelerated speed than those cultured on the scaffolds. On day 3 and day 7, UC-MSCs cultured on the 3D-printed HA showed an almost similar proliferation rate to those cultured on a plastic culture plate. However, from day 14 onward, UC-MSCs cultured on a coated 3D-printed HA had a significantly higher percentage of cell viability than UC-MSCs cultured on a plastic culture plate (*P* < 0.05). The results indicated that the scaffolds were biocompatible with both BM-MSCs and UC-MSCs, with the cell proliferation and expansion more noticeable on the coated 3D-printed HA than on 3D-printed HA.

### Alkaline phosphatase activity

**Figure 7:**
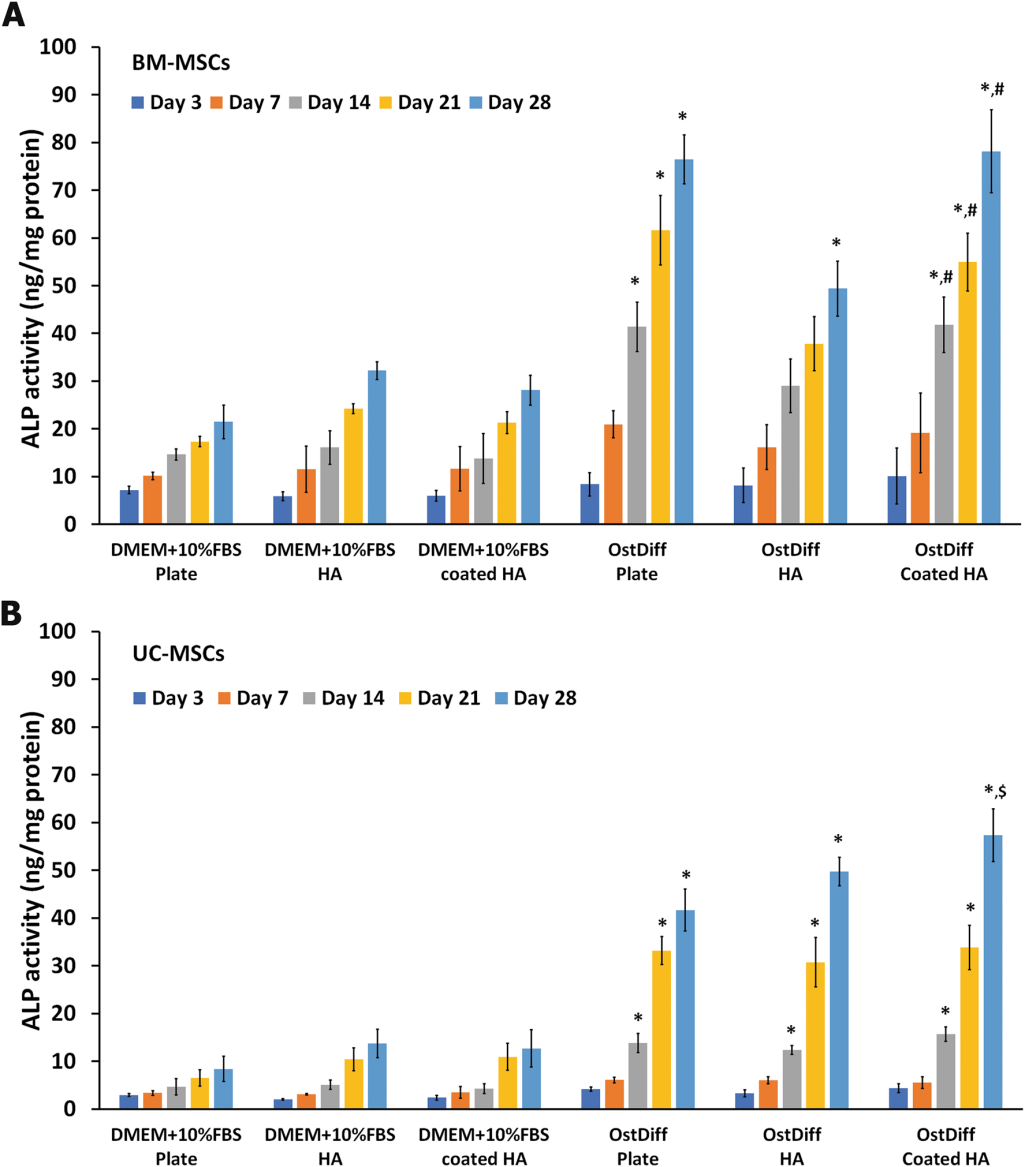
Alkaline phosphatase activity of BM-MSCs (**A**) and UC MSCs (**B**) cultured on the 3D-printed HA (HA) and coated 3D-printed HA (coated HA) compared with those cultured on a plastic culture plate at days 3, 7, 14, 21, and 28. N = 3, *P < 0.05, compared to MSCs cultured with DMEM + 10%FBS. ^#^*P* < 0.05, compared to MSCs cultured with osteogenic differentiation medium on 3D-printed HA. ^$^*P* < 0.05 compared to MSCs cultured with osteogenic differentiation medium on a plastic culture plate.

The quantitative ALP activity assay evaluated the ability of 3D-printed HA and coated 3D-printed HA to support and facilitate osteogenic differentiation of BM-MSCs and UC-MSCs. The ALP activity of BM-MSCs cultured with osteogenic induction medium on 3D-printed HA and coated 3D-printed HA showed a gradual and significant increase compared to those cultured in the complete DMEM medium, regardless of the supporting substrates (Fig. 7A). The highest ALP activity was observed on day 28 in the cells cultured on both the scaffolds and the plastic culture plates. For almost all the observed days, the ALP activity of BM-MSCs cultured on the plastic culture plate was higher than that cultured on the scaffolds except for BM-MSCs cultured on coated 3D-printed HA scaffolds on day 28 (Fig. 7A). Interestingly, the ALP activity of BM-MSCs cultured on coated 3D-printed HA scaffold was higher than that cultured on the 3D-printed HA scaffold at every time point; however, a significant difference was observed only on day 28 (*P* < 0.05).

Similarly, UC-MSCs cultured with osteogenic differentiation medium on both types of scaffolds and the plastic culture plate had higher ALP activity than the cells cultured with completed DMEM medium (Fig. 7B). The ALP activity of UC-MSCs cultured with osteogenic differentiation medium increased steadily until day 28 in the scaffolds and plastic culture plate. ALP activity of such UC-MSCs had significantly increased from day 14 onwards (Fig. 7B). On day 28, UC-MSCs cultured with osteogenic differentiation medium on the 3D-printed HA and coated 3D-printed HA scaffolds had a significantly greater ALP activity than those cultured on the plastic culture plate (*P* < 0.05).

### The expression level of osteogenic genes

**Figure 8:**
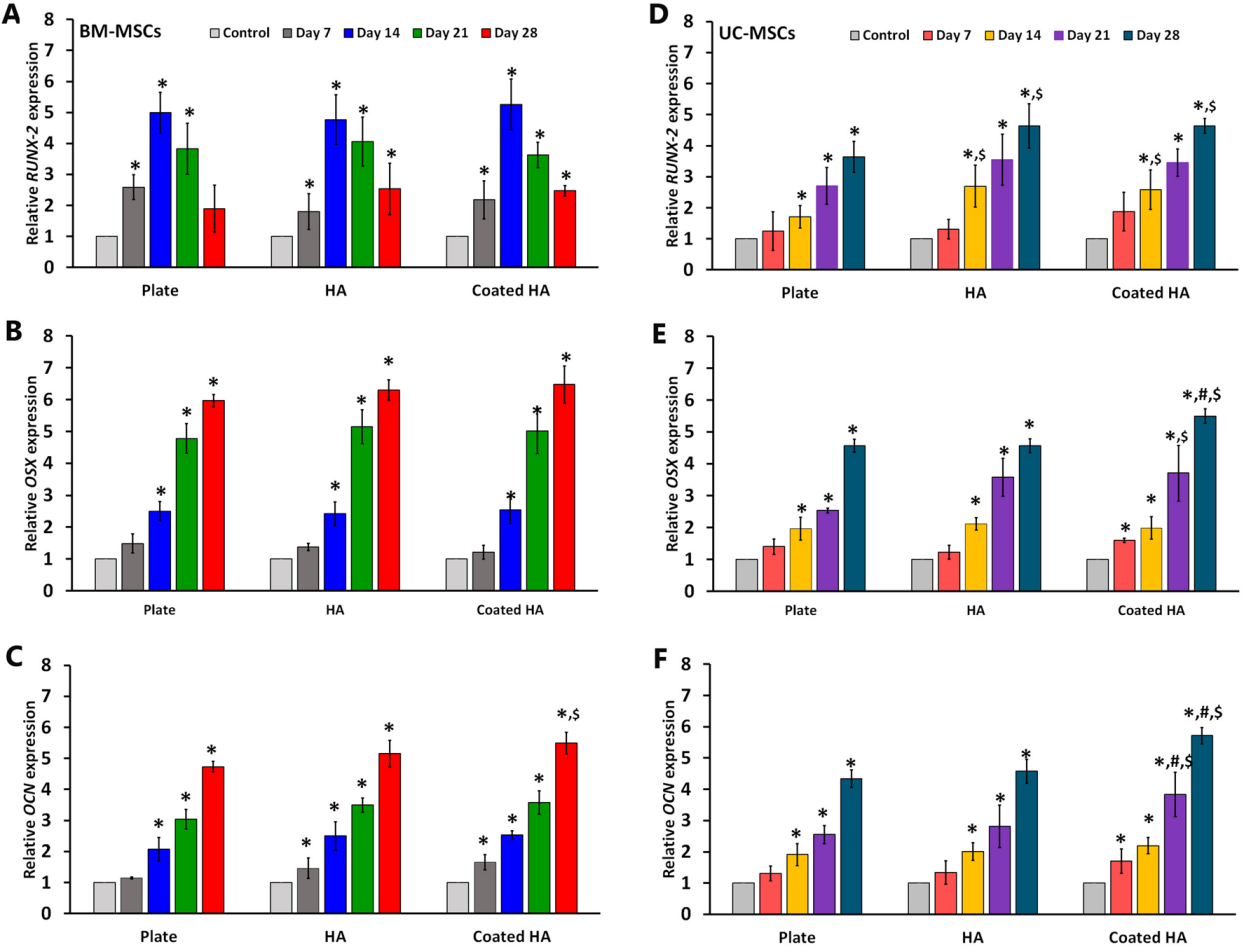
Real-time RT-PCR illustrates the expressions of osteogenic markers, *RUNX-2*, osterix (*OSX*), and osteocalcin (*OCN*) in BM-MSCs (**A**, **B**, **C**) and UC-MSCs (**D**, **E**, **F**) during cultured on a plastic culture plate, the 3D-printed HA (HA) and coated 3D-printed HA (coated HA). MSCs cultured in complete DMEM medium at day 0 serve as the control. *Statistically significant data with *P* < 0.05 compared to control. N = 3, ^#^*P* < 0.05 compared to MSCs cultured on 3D-printed HA at the same time point. ^$^*P* < 0.05 compared to MSCs cultured on a plastic culture plate at the same time point.

To compare the osteogenic differentiation potential of BM-MSCs and UC-MSCs cultured on the 3D-printed HA and coated 3D-printed HA with those cultured in a plastic culture plate, the expression of osteogenic genes was monitored every week using quantitative real-time PCR. The results indicated that the expression of *RUNX-2* in BM-MSCs cultured on the 3D-printed HA and coated 3D-printed HA steadily increased and reached its highest level on day 14 before gradually decreasing on day 21 and day 28. A similar progression of *RUNX-2* expression was observed in BM-MSCs cultured on a plastic culture plate (Fig. 8A). It is worth noting that, on day 14, BM-MSCs cultured on the coated 3D-printed HA scaffolds had a significantly higher expression level of *RUNX-2* than BM-MSCs cultured on the 3D-printed HA scaffold. In contrast to *RUNX-2*, both OSX and OCN expression levels in BM-MSCs cultured on the 3D-printed HA and coated 3D-printed HA gradually increased throughout the entire culture and reached the highest points at the end of the culture period (Fig. 8B, C). The BM-MSCs cultured on the 3D-printed HA and coated 3D-printed HA showed higher *OSX* and *OCN* expressions than the cells cultured on a plastic culture plate; however, a significant difference in *OCN* expression was observed only on day 28 (*P* < 0.05).

For UC-MSCs, *RUNX-2* expression in cells cultured on the 3D-printed HA and coated 3D-printed HA was progressively increased and reached the highest level on day 28. UC-MSCs cultured in an osteogenic induction medium on a plastic culture plate showed a similar pattern of *RUNX-2* expression to MSCs cultured on the scaffolds (Fig. 8D). Remarkably, UC-MSCs cultured on the 3D-printed HA and coated 3D-printed HA scaffolds had significantly higher *RUNX-2* expression than the cells cultured on a plastic culture plate (*P* < 0.05). In addition, the expression levels of *OSX* and *OCN* in UC-MSCs cultured on the scaffolds and plastic culture plate had significantly increased in a time-dependent manner compared to those cultured in the complete DMEM medium (*P* < 0.05). Notably, the most robust *OSX* expression was found in UC-MSCs cultured on the coated 3D-printed HA (Fig. 8E). Also, UC-MSCs cultured on the coated 3D-printed HA showed significantly upregulated expression of OCN on day 28 compared to the cells cultured on the 3D-printed HA (Fig. 8F).

### The expression of collagenous and non-collagenous proteins

**Figure 9:**
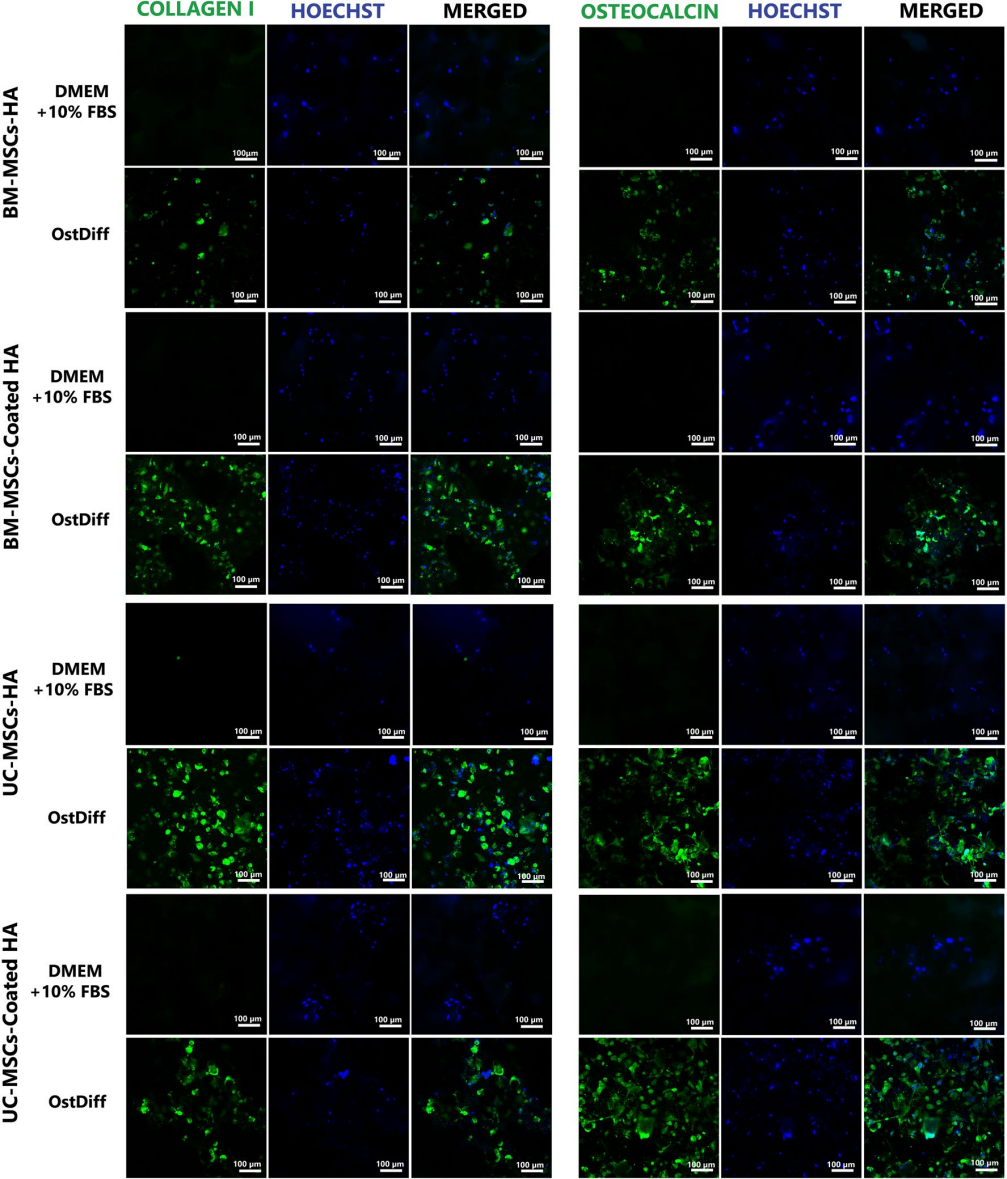
Immunofluorescent micrograph illustrated the expression of osteocalcin and collagen I in BM-MSCs and UC-MSCs cultured on the 3D-printed HA (HA) and coated 3D-printed HA (coated HA) for 21 days.

Immunofluorescence staining was performed on day 21 after osteogenic induction to examine the expression of osteogenic protein markers, including osteocalcin and collagen I in BM-MSCs and UC-MSCs cultured on the 3D-printed HA and coated 3D-printed HA. The results showed that BM-MSCs cultured on the 3D-printed HA were positive for osteocalcin staining, similar to those cultured on coated 3D-printed HA. In addition, BM-MSCs cultured on the 3D-printed HA had an intense expression of collagen I similar to those cultured on coated 3D-printed HA (Fig. 9). Correspondingly, UC-MSCs cultured on the 3D-printed HA were positive for osteocalcin staining similar to those cultured on coated 3D-printed HA. In addition, UC-MSCs cultured on the 3D-printed HA had an intense expression of collagen I similar to those cultured on coated 3D-printed HA (Fig. 9).

## Discussion

As the use of mesenchymal stem cells (MSCs) for bone repair increases rapidly, it is necessary to determine which tissues may provide the most suitable sources and to verify the specific potencies of MSCs. The limited availability of bone marrow, the primary source of MSCs for clinical applications, necessitates a lengthy amplification process. Long-term culture affects the biological properties of MSCs and diminishes their proliferation and differentiation potentials^46^. In allogeneic transplantation, the therapeutic efficacy is associated with the quantity and number of doses of MSCs being administered^47^. Therefore, alternative MSCs from more readily available sources should be more convenient for bone repair. MSCs can be isolated from the umbilical cord, a discarded biological sample after delivery^48^. The UC-MSCs share similar characteristics with BM-MSCs through having a similar pattern of cell surface markers and differentiation potentials^28,49,50^. However, their osteogenic differentiation potential is less pronounced than BM-MSCs^41^. Manipulating culture conditions, such as introducing specific growth factors, could result in more efficient MSC expansion and osteogenic differentiation^28,29^. Therefore, UC-MSCs are promising stem cells for allogeneic bone tissue repair. If proven feasible for clinical application, UC-MSCs have an advantage as they can be prepared in large quantities and as an off-the-shelf product with faster tissue processing than using autologous BM-MSCs.

According to the standard characterization of MSCs, the clonal cells must adhere to a plastic culture plate, express cluster of differentiation (CD) markers, including CD73, CD90, and CD105, and can differentiate into adipogenic, chondrogenic as well as osteogenic lineages in vitro^38^. This study examined the characteristics of BM-MSCs and UC-MSCs by observing their morphology in plastic culture plate, analyzed the expression of cell surface markers using flow cytometry, and their potential to differentiate into adipocytes, chondrocytes, and osteoblasts. Both BM-MSCs and UC-MSCs exhibited spindle shape when grown in a plastic culture plate, expressed a similar cell surface marker pattern. They could differentiate into osteoblasts, chondrocytes, and adipocytes. In addition, this study also demonstrated that BM-MSCs and UC-MSCs had a high cell proliferation rate. The doubling times steadily increased from P2 to P8. The overall decrease in cell proliferation in the case of BM-MSCs observed after the eighth passage is consistent with previous studies showing that MSCs have a limited lifespan and enter senescence after a certain number of cell divisions^51^. Remarkably, UC-MSCs display a higher expansion potential than BM-MSCs; BM-MSCs could actively expand in less than 10 passages, and UC-MSCs could actively expand up to 20 passages before losing their proliferative capacity after passage 20^52^. The simplicity of cell isolation and amplification allows for efficient UC-MSC manufacture.

When the immunophenotype was analyzed by flow cytometry, it was found that both BM-MSCs and UC-MSCs did not express MSC exclusion markers, CD34 and CD45. On the other hand, these MSCs expressed the recommended MSC markers, CD73, CD90, and CD105^53^. These MSCs have also been shown to maintain their adipogenic, chondrogenic, and osteogenic differentiation ability after amplification. However, the differentiation abilities were variable depending on the cell source.

To enhance the efficiency of MSCs in bone repair, this study attempted to develop a 3D cultured system that better supported the proliferation and osteogenic differentiation of these MSCs. For successful bone tissue engineering, using a scaffold may be beneficial to maintain the transplanted MSCs in a specific location. Moreover, a 3D scaffold acts as an extracellular matrix that supports cell adhesion, proliferation, differentiation, spreading, and communication^54^. Hydroxyapatite is a good candidate for bone repair among the various calcium phosphate ceramics because it contains similar chemical compositions to natural hydroxyapatite in bone tissue^55^. It also has excellent biocompatibility and osteoconductive properties. Bone grafting using hydroxyapatite results in direct chemical bonding of hydroxyapatite to the bone^56^. Hydroxyapatite is used as a single material or in combination with other materials, such as a coating agent for bone graft^57^ and a matrix for drug delivery systems targeting bone tissue repair^58^. A customized scaffold with the desirable anatomical contour can be manufactured via 3D printing as the technique allows simple, fast, accurate, and several tests to be conducted on the biomaterial. Scanning electron microscopy illustrated the porous microstructure of the 3D-printed HA and coated 3D-printed HA, enabling the transportation of nutrients and metabolites into the bone tissue. Highly porous scaffolds are often used in bone tissue repair to mimic the porosity of the trabecular bone^59^. The different surface topography between the 3D-printed HA and coated 3D-printed HA might affect the interaction of MSCs on the scaffolds. A rough surface stimulates the spreading and proliferation of osteoblast-liked cells^60^. Therefore, both the inner and surface characteristics of the scaffolds play essential parts in the success of the therapeutic application.

Both BM-MSCs and UC-MSCS cultured on the 3D-printed HA and coated 3D-printed HA could be microscopically identified by staining with fluorescent phalloidin, which binds to F-actin and indicates the presence and change of the cell cytoskeleton^61^. The appearance of F-actin stress fibers extending across the entire cytoplasm indicates proper attachment and affinity of MSCs to the surfaces of the scaffolds. One of the most important aspects of bone tissue engineering is the interactions between the cells and the biomaterial that supports them. The biocompatibility of the scaffolds was further evaluated by a prolonged culture period until 28 days. Scanning electron microscopy showed many MSC colonies appearing as sheets of elongated cells with extended filopodia on the surface of the scaffolds. This indicated that both the 3D-printed HA and coated 3D-printed HA could support the proliferation of BM-MSCs and UC-MSCs. Therefore, these scaffolds could be suitable candidate materials for bone tissue engineering.

Bone regeneration is known to occur by recruiting MSCs to the injury site. After that, cell proliferation, osteoblastic differentiation, and intramembranous ossification happen. The ability of the cells to proliferate on the scaffolds implies they can mimic the native extracellular matrix in supporting cell growth and differentiation through the effective linkage between the cells and scaffold^62^. The data from this study revealed that MSCs cultured on both scaffolds had higher cell proliferation rates than those cultured on a plastic culture plate, especially in UC-MSCs. A previous study reported that particle sizes in HA also influenced cell proliferation: MSCs cultured on smaller HA particles exhibited a higher proliferation rate than larger HA particles^63^. Both BM-MSCs and UC-MSCs significantly increased their proliferation potential when cultured on the coated 3D-printed HA. The result suggested that the coated 3D-printed HA, which contained additional OCP on the surface, promoted better cell survival and adhesion. Previous studies reported that OCP coatings remarkably enhanced cell proliferation and ALP activity of MC3T3-E1 cells^64–66^. Therefore, it was suggested that OCP could control the environment during the conversion to HA under physiological conditions, which increased biological activity and bone regeneration enhancement^22^.

ALP is a primary phenotypic indicator secreted by osteoblasts. Upregulation of ALP occurs during early osteogenesis^67^. Therefore, the expression of ALP is an early marker of osteogenic differentiation of human MSCs^68^. Assessing the levels of ALP activity in BM-MSCs and UC-MSCs cultured on the 3D-printed HA and coated 3D-printed HA helped validate the differentiation of the MSCs towards the osteogenic lineage. The observed result revealed that BM-MSCs and UC-MSCs cultured on the coated 3D-printed HA had a significantly increased level of ALP activity compared to those cultured on the 3D-printed HA scaffolds. Furthermore, this event was more pronounced for BM-MSCs than UC-MSCs. Overall, the data verified that the coated 3D-printed HA could support osteogenic differentiation, as indicated by enhanced ALP activity, better than the 3D-printed HA.

In agreement with ALP activity, MSCs cultured on the coated 3D-printed HA had a higher level of osteogenic gene expression than those cultured on the 3D-printed HA. Statistically, significant differences were observed in the case of UC-MSCs. By comparison, both MSCs cultured on both types of scaffolds had a higher expression of most of the osteogenic genes than those cultured on plastic culture plates. A previous study reported that HA could induce the activity of osteoblasts, which increases the synthesis of a new bone matrix in bone defects^55^ by upregulating *RUNX-2* expression^69^. During osteogenesis, osteoblasts differentiate from their precursors through the modulations of several transcription factors, including the master transcription factor *RUNX-2* and its downstream *OSX*. These two are critical transcription factors that play crucial roles in the cell-fate decision process through which MSCs become osteoblasts^70^. *RUNX-2* is one of the most common markers indicative of osteoblastic differentiation, especially at an early stage. The expression of *RUNX-2* decreases over time during osteoblast differentiation^71^.

On the other hand, the overexpression of *RUNX-2* in the late stage of osteoblast differentiation inhibits osteoblast maturation, decreases bone mass, and causes osteopenia and bone fracture^72^. It was observed that the expression level of *RUNX-2* in BM-MSCs was highest on day 14 and gradually decreased until day 28, whereas the expression level of *RUNX-2* in UC-MSCs was progressively increased until day 28. It might be possible that BM-MSCs differentiated into the osteoblast more efficiently and quickly than UC-MSCs^73^; therefore, the peak of *RUNX-2* expression in BM-MSCs was observed on day 14, while the peak of *RUNX-2* expression in UC-MSCs was observed on day 28. *OSX* is a downstream factor of *RUNX-2*^74^, and its expression induces the differentiation of the early osteoblasts into the mature ones and finally into osteocytes during bone formation^75^. Inactivation of *OSX* in the postnatal period caused defects in osteoblasts' function, which diminished bone formation^76^. Together with *RUNX-2*, *OSX* is responsible for expressing osteoblast proteins, including ALP, collagen, and non-collagenous proteins^77^. In addition to *RUNX-2*, HA also induced *OSX* expression, which caused osteoblastic differentiation of osteoblast progenitor cells^78^. *OCN* is a γ-carboxyglutamate protein secreted almost exclusively by osteoblasts. It is the most abundant non-collagenous protein in bone tissue that confers high affinity to the bone hydroxyapatite matrix^79^. It is also responsible for the synthesis and mineralization of the bone matrix during the development of the skeleton. Therefore, the expression level of *OCN* in osteoblasts is a marker of mineral deposition^79^. These factors are commonly used as markers in studies involving osteoblastic differentiation, including those related to the use of HA for bone regeneration. It has been reported that HA induced osteoblastic differentiation by increasing the expression of osteogenic transcription factors^55^. In agreement with gene expression, the protein analysis revealed that specific osteogenic markers, including non-collagenous protein, OCN, and collagenous protein, COL1, were expressed in MSCs cultured on both types of 3D-printed HA scaffolds. The data suggest that BM-MSCs and UC-MSCs cultured on 3D-printed HA and coated 3D-printed HA could be differentiated into osteoblasts.

The data obtained from this study increase the understanding of the molecular mechanisms of HA as a biomaterial for bone tissue engineering and provides a better design of HA-based scaffolds to be exploited in bone tissue regeneration. Notably in the case of the coated 3D-printed HA. It is possible that the calcium phosphate coating might better support in vitro osteogenic differentiation of MSCs and improve in vitro osteogenic formation. This is supported by the finding that biomaterial containing calcium phosphate moieties has been shown to promote the osteogenic differentiation of human MSCs in vitro^80^. Taken together, our study demonstrated the benefit and suitability of the 3D-printed HA and coating 3D-printed HA for possible applications in bone regeneration and treating bone diseases.

## Conclusion

This study demonstrated the potential of UC-MSCs and BM-MSCs in bone tissue engineering. Both 3D-printed HA and calcium phosphate-coated 3D-printed HA supported the proliferation and osteogenic differentiation of BM-MSCs and UC-MSCs. Remarkably, the coated 3D-printed HA displayed considerably higher biocompatibility than the 3D-printed HA. Nevertheless, both 3D-printed HA and coated 3D-printed HA showed good potential as biomaterials that could be constructed into novel scaffolds for bone tissue repair.

## Data Availability

All data generated or analyzed during this study are included in this published article.

## Abbreviations

ALP: Alkaline phosphatase
BM-MSCs: Bone marrow-derived mesenchymal stem cells
CD: Cluster of differentiation
DAPI: 4, 6‐Diamidino‐2‐phenylindole
DMEM: Dulbecco's Modified Eagle's Medium
EDTA: Ethylenediamine tetraacetic acid
FBS: Fetal bovine serum
FITC: Fluorescein isothiocyanate
GAPDH: Glyceraldehyde-3-phosphate dehydrogenase
HA: Hydroxyapatite scaffold
MSCs: Mesenchymal stem cells
OCN: Osteocalcin
OSX: Osterix
PBS: Phosphate buffered saline
PE: Phycoerythrin
pNPP: p-Nitrophenyl phosphate
qRT-PCR: Quantitative real-time polymerase chain reaction
RUNX-2: Runt-related transcription factor 2
SEM: Standard error of means
UC-MSCs: Umbilical cord-derived mesenchymal stem cells

## References

[1] Polo-Corrales L, Latorre-Esteves M, Ramirez-Vick JE (2014). Scaffold design for bone regeneration. J. Nanosci. Nanotechnol..

[2] Porter JR, Ruckh TT, Popat KC (2009). Bone tissue engineering: A review in bone biomimetics and drug delivery strategies. Biotechnol. Prog..

[3] Li J (2011). Repair of rat cranial bone defects with nHAC/PLLA and BMP-2-related peptide or rhBMP-2. J. Orthop. Res. Off. Publ. Orthop. Res. Soc..

[4] Lane JM, Tomin E, Bostrom MP (1999). Biosynthetic bone grafting. Clin. Orthop. Relat. Res..

[5] Oryan A, Alidadi S, Moshiri A, Maffulli N (2014). Bone regenerative medicine: Classic options, novel strategies, and future directions. J. Orthop. Surg. Res..

[6] Qi H (2016). Bioactivity assessment of PLLA/PCL/HAP electrospun nanofibrous scaffolds for bone tissue engineering. Life Sci..

[7] Burg KJ, Porter S, Kellam JF (2000). Biomaterial developments for bone tissue engineering. Biomaterials.

[8] Motamedian SR (2017). Response of dental pulp stem cells to synthetic, allograft, and xenograft bone scaffolds. Int. J. Periodontics Restorative Dent..

[9] Bose S, Roy M, Bandyopadhyay A (2012). Recent advances in bone tissue engineering scaffolds. Trends Biotechnol..

[10] Birhanu G, Akbari JH, Seyedjafari E, Zandi-Karimi A, Dusti TM (2018). An improved surface for enhanced stem cell proliferation and osteogenic differentiation using electrospun composite PLLA/P123 scaffold. Artif. Cells Nanomed. Biotechnol..

[11] Xu T, Sheng L, He L, Weng J, Duan K (2020). Enhanced osteogenesis of hydroxyapatite scaffolds by coating with BMP-2-loaded short polylactide nanofiber: A new drug loading method for porous scaffolds. Regen. Biomater..

[12] Lin L, Chow KL, Leng Y (2009). Study of hydroxyapatite osteoinductivity with an osteogenic differentiation of mesenchymal stem cells. J. Biomed. Mater. Res. A.

[13] Krishnamurithy G (2013). A review on hydroxyapatite-based scafolds as a potential bone graft substitute for bone tissue engineering application. JUMMEC.

[14] Quan WXZ, Bin W, Wei H (2013). Effects of microwave sintering on the properties of porous hydroxyapatite scaffolds. Ceram. Int..

[15] Yunzhi YDD, Joo LO (2005). Protein adsorption and osteoblast precursor cell attachment to hydroxyapatite of different crystallinities. Int. J. Oral Maxillofacial Implants.

[16] Suwanprateeb J, Suvannapruk W, Wasoontararat K (2010). Low temperature preparation of calcium phosphate structure via phosphorization of 3D-printed calcium sulfate hemihydrate based material. J. Mater. Sci. Mater. Med..

[17] Suwanprateeb J, Thammarakcharoen F (2017). Influence of surface pretreatment on the coating quantity and properties of nanostructured octacalcium phosphate on commercially pure titanium. Sci. Rep..

[18] Drevelle O, Faucheux N (2013). Biomimetic materials for controlling bone cell responses. Front. Biosci. (Schol Ed).

[19] Liu Y, Wu G, de Groot K (2010). Biomimetic coatings for bone tissue engineering of critical-sized defects. J. R. Soc. Interface.

[20] Thammarakcharoen F, Suwanprateeb J (2017). Effect of process parameters on biomimetic deposition of calcium phosphate on 3D printed hydroxyapatite. Key Eng. Mater..

[21] Habibovic P, van der Valk CM, van Blitterswijk CA, De Groot K, Meijer G (2004). Influence of octacalcium phosphate coating on osteoinductive properties of biomaterials. J. Mater. Sci. Mater. Med..

[22] Suzuki O, Anada T (2013). Synthetic octacalcium phosphate: A possible carrier for mesenchymal stem cells in bone regeneration. Annu. Int. Conf. IEEE Eng. Med. Biol. Soc..

[23] Yang Y (2015). Bioinspired porous octacalcium phosphate/silk fibroin composite coating materials prepared by electrochemical deposition. ACS Appl. Mater. Interfaces.

[24] Thammarakcharoen F, Suwanprateeb J (2020). BMP-2 incorporation into 3D printed porous hydroxyapatite by rapid biomimetic co-precipitation technique using accelerated calcium phosphate solution. Chiang Mai J. Sci..

[25] Su P (2018). Mesenchymal stem cell migration during bone formation and bone diseases therapy. Int. J. Mol. Sci..

[26] Centeno, C. J. & Faulkner, S. J. in *Stem Cells and Cancer Stem Cells* Vol. 1 (ed M.A. Hayat) Ch. 21, 173–179 (2012).

[27] Barry FP (2003). Biology and clinical applications of mesenchymal stem cells. Birth Defects Res. C Embryo Today.

[28] Marupanthorn K, Tantrawatpan C, Kheolamai P, Tantikanlayaporn D, Manochantr S (2017). Bone morphogenetic protein-2 enhances the osteogenic differentiation capacity of mesenchymal stromal cells derived from human bone marrow and umbilical cord. Int. J. Mol. Med..

[29] Manochantr S (2017). The effects of BMP-2, miR-31, miR-106a, and miR-148a on osteogenic differentiation of MSCs derived from amnion in comparison with MSCs derived from the bone marrow. Stem Cells Int..

[30] Zhou S (2008). Age-related intrinsic changes in human bone-marrow-derived mesenchymal stem cells and their differentiation to osteoblasts. Aging Cell.

[31] Stenderup K, Justesen J, Clausen C, Kassem M (2003). Aging is associated with decreased maximal life span and accelerated senescence of bone marrow stromal cells. Bone.

[32] Kassem M, Marie PJ (2011). Senescence-associated intrinsic mechanisms of osteoblast dysfunctions. Aging Cell.

[33] Erices A, Conget P, Minguell JJ (2000). Mesenchymal progenitor cells in human umbilical cord blood. Br J Haematol.

[34] Campagnoli C (2001). Identification of mesenchymal stem/progenitor cells in human first-trimester fetal blood, liver, and bone marrow. Blood.

[35] In ‘t Aanker PS (2004). Isolation of mesenchymal stem cells of fetal or maternal origin from human placenta. Stem Cells.

[36] Soncini M (2007). Isolation and characterization of mesenchymal cells from human fetal membranes. J. Tissue Eng. Regen. Med..

[37] Portmann-Lanz CB (2006). Placental mesenchymal stem cells as potential autologous graft for pre- and perinatal neuroregeneration. Am. J. Obstet. Gynecol..

[38] Pittenger MF (1999). Multilineage potential of adult human mesenchymal stem cells. Science.

[39] Manochantr S (2013). Immunosuppressive properties of mesenchymal stromal cells derived from amnion, placenta, Wharton's jelly and umbilical cord. Intern. Med. J..

[40] Matas J (2019). Umbilical cord-derived mesenchymal stromal cells (MSCs) for knee osteoarthritis: Repeated MSC dosing is superior to a single MSC dose and to hyaluronic acid in a controlled randomized phase I/II trial. Stem Cells Transl. Med..

[41] Shen C, Yang C, Xu S, Zhao H (2019). Comparison of osteogenic differentiation capacity in mesenchymal stem cells derived from human amniotic membrane (AM), umbilical cord (UC), chorionic membrane (CM), and decidua (DC). Cell Biosci..

[42] Kmiecik G, Spoldi V, Silini A, Parolini O (2015). Current view on osteogenic differentiation potential of mesenchymal stromal cells derived from placental tissues. Stem Cell Rev. Rep..

[43] Heo JS, Choi Y, Kim HS, Kim HO (2016). Comparison of molecular profiles of human mesenchymal stem cells derived from bone marrow, umbilical cord blood, placenta and adipose tissue. Int. J. Mol. Med..

[44] Marupanthorn K, Tantrawatpan C, Kheolamai P, Tantikanlayaporn D, Manochantr S (2021). MicroRNA treatment modulates osteogenic differentiation potential of mesenchymal stem cells derived from human chorion and placenta. Sci. Rep..

[45] Barlow S (2008). Comparison of human placenta- and bone marrow-derived multipotent mesenchymal stem cells. Stem Cells Dev..

[46] Kozlowska U (2019). Similarities and differences between mesenchymal stem/progenitor cells derived from various human tissues. World J. Stem Cells.

[47] Lutton BV (2010). Approaches to avoid immune responses induced by repeated subcutaneous injections of allogeneic umbilical cord tissue-derived cells. Transplantation.

[48] Marupanthorn K, Tantrawatpan C, Tantikanlayaporn D, Kheolamai P, Manochantr S (2015). The effects of TNF-alpha on osteogenic differentiation of umbilical cord derived mesenchymal stem cells. J. Med. Assoc. Thai.

[49] Baksh D, Yao R, Tuan RS (2007). Comparison of proliferative and multilineage differentiation potential of human mesenchymal stem cells derived from umbilical cord and bone marrow. Stem Cells.

[50] Thaweesapphithak S (2019). Human serum enhances the proliferative capacity and immunomodulatory property of MSCs derived from human placenta and umbilical cord. Stem Cell Res. Ther..

[51] Wagner W (2009). Aging and replicative senescence have related effects on human stem and progenitor cells. PLoS ONE.

[52] Fabre H (2019). Characterization of different sources of human MSCs expanded in serum-free conditions with quantification of chondrogenic induction in 3D. Stem Cells Int..

[53] Dominici M (2006). Minimal criteria for defining multipotent mesenchymal stromal cells. The International Society for Cellular Therapy position statement. Cytotherapy.

[54] Liu Y (2013). Contrasting effects of vasculogenic induction upon biaxial bioreactor stimulation of mesenchymal stem cells and endothelial progenitor cells cocultures in three-dimensional scaffolds under in vitro and in vivo paradigms for vascularized bone tissue engineering. Tissue Eng. Part A.

[55] Khotib J (2021). Signaling pathway and transcriptional regulation in osteoblasts during bone healing: Direct involvement of hydroxyapatite as a biomaterial. Pharmaceuticals (Basel).

[56] Scalera F, Palazzo B, Barca A, Gervaso F (2020). Sintering of magnesium-strontium doped hydroxyapatite nanocrystals: Towards the production of 3D biomimetic bone scaffolds. J. Biomed. Mater. Res. Part A.

[57] Samirah BAS, Mahyudin F, Khotib J (2021). Fabrication and characterization of bovine hydroxyapatite-gelatin-alendronate scaffold cross-linked by glutaraldehyde for bone regeneration. J. Basic Clin. Physiol. Pharmacol..

[58] Budiatin AS (2021). Bovine hydroxyapatite-based bone scaffold with gentamicin accelerates vascularization and remodeling of bone defect. Int. J. Biomater..

[59] Rather HA, Jhala D, Vasita R (2019). Dual functional approaches for osteogenesis coupled angiogenesis in bone tissue engineering. Mater. Sci. Eng. C Mater. Biol. Appl..

[60] Osathanon T, Bespinyowong K, Arksornnukit M, Takahashi H, Pavasant P (2011). Human osteoblast-like cell spreading and proliferation on Ti-6Al-7Nb surfaces of varying roughness. J. Oral Sci..

[61] Li S (2014). Inhibition of osteogenic differentiation of mesenchymal stem cells by copper supplementation. Cell Prolif..

[62] Gandhimathi C (2013). Mimicking nanofibrous hybrid bone substitute for mesenchymal stem cells differentiation into osteogenesis. Macromol. Biosci..

[63] Weissenboeck M (2006). Particle size of hydroxyapatite granules calcified from red algae affects the osteogenic potential of human mesenchymal stem cells in vitro. Cells Tissues Organs.

[64] Kalinichenko SG, Matveeva NY, Kostiv RY, Edranov SS (2021). The effect of calcium phosphate biodegradable coatings of titanium implants on cell differentiation and apoptosis in rat bone tissue after experimental fracture. Biomed. Mater. Eng..

[65] Jiang P (2015). Effect of octacalcium-phosphate-modified micro/nanostructured titania surfaces on osteoblast response. ACS Appl. Mater. Interfaces.

[66] Fan L (2021). Surface properties of octacalcium phosphate nanocrystals are crucial for their bioactivities. ACS Omega.

[67] Butterworth PJ (1983). Alkaline phosphatase. Biochemistry of mammalian alkaline phosphatases. Cell Biochem. Funct..

[68] Kim YH, Yoon DS, Kim HO, Lee JW (2012). Characterization of different subpopulations from bone marrow-derived mesenchymal stromal cells by alkaline phosphatase expression. Stem Cells Dev..

[69] Mao L (2015). Effect of micro-nano-hybrid structured hydroxyapatite bioceramics on osteogenic and cementogenic differentiation of human periodontal ligament stem cell via Wnt signaling pathway. Int. J. Nanomed..

[70] Kawane T (2018). Runx2 is required for the proliferation of osteoblast progenitors and induces proliferation by regulating Fgfr2 and Fgfr3. Sci. Rep..

[71] Beck GR (2003). Inorganic phosphate as a signaling molecule in osteoblast differentiation. J. Cell Biochem..

[72] Liu W (2001). Overexpression of Cbfa1 in osteoblasts inhibits osteoblast maturation and causes osteopenia with multiple fractures. J. Cell Biol..

[73] Kouroupis D (2013). Assessment of umbilical cord tissue as a source of mesenchymal stem cell/endothelial cell mixtures for bone regeneration. Regen. Med..

[74] Cao Y (2005). Osterix, a transcription factor for osteoblast differentiation, mediates antitumor activity in murine osteosarcoma. Cancer Res..

[75] Sinha KM, Zhou X (2013). Genetic and molecular control of osterix in skeletal formation. J. Cell Biochem..

[76] Baek WY, de Crombrugghe B, Kim JE (2010). Postnatally induced inactivation of Osterix in osteoblasts results in the reduction of bone formation and maintenance. Bone.

[77] Licini C, Vitale-Brovarone C, Mattioli-Belmonte M (2019). Collagen and non-collagenous proteins molecular crosstalk in the pathophysiology of osteoporosis. Cytokine Growth Factor Rev..

[78] Baek WY, Kim JE (2011). Transcriptional regulation of bone formation. Front. Biosci. (Schol Ed).

[79] Manolagas SC (2020). Osteocalcin promotes bone mineralization but is not a hormone. PLoS Genet..

[80] Shih YR (2014). Calcium phosphate-bearing matrices induce osteogenic differentiation of stem cells through adenosine signaling. Proc. Natl. Acad. Sci. U S A.

